# Modeling Phenotypic Trait Variation and Plasticity in *Elymus elymoides* to Guide Climate‐Informed Seed Transfer

**DOI:** 10.1111/eva.70211

**Published:** 2026-03-06

**Authors:** Francis F. Kilkenny, Jeffrey E. Ott, Elizabeth A. Leger, Richard C. Johnson, Matthew E. Horning, J. Bradley St. Clair

**Affiliations:** ^1^ USDA Forest Service Rocky Mountain Research Station Boise USA; ^2^ Department of Biology University of Nevada Nevada USA; ^3^ USDA Agricultural Research Service Pullman Washington USA; ^4^ USDA Forest Service International Programs – Office of the Chief Bend Oregon USA; ^5^ USDA Forest Service Pacific Northwest Research Station Corvallis Oregon USA

**Keywords:** common garden study, fixed‐boundary seed zones, focal‐point seed transfer models, multivariate regression tree modeling, random forests, restoration seed sourcing

## Abstract

Information on climate‐associated phenotypic variation is essential for sourcing seed that matches restoration site conditions. Spatially explicit seed transfer models can effectively deliver this information. However, standard modeling approaches often do not provide flexibility for practical considerations and may not capture highly complex trait‐climate associations. We characterized climate‐associated variation in growth, reproduction, morphology, phenology, and survival across 98 source populations at 3 common gardens for the grass *Elymus elymoides* (bottlebrush squirreltail), an important restoration species in the Intermountain Region of the western USA. We developed fixed‐boundary seed zones and focal‐point seed transfer models using non‐standard methods (regression trees and random forests). In general, source populations with larger plant sizes and later flowering originated from cooler and wetter or milder climates than those with smaller sizes and earlier flowering, though some associations were more complex. Populations from milder climates also had higher trait plasticity than populations from other climates, except for plasticity in seed maturation, which was highest in populations from warmer and drier climates. Seed zones identified through our approach consisted of three major zones with 2–7 subzones each (13 seed zones in total). Two subspecies groups had distinct trait‐climate associations, and separate seed zone models were developed for each. Our modeling approach provides a hierarchical structure that partitions predictor variables based on their importance. This doubles as a prioritization framework that assists in navigating trade‐offs between risk avoidance and practical constraints by explicitly defining how zones can be combined or subdivided in response to user needs. Our approach also captures trait‐climate association nuances missed by standard approaches, increasing the precision of our focal‐point seed transfer zones. Our findings emphasize the multifaceted nature of trait‐climate associations and highlight the importance of seed transfer modeling to seed‐sourcing decisions in a time of global change.

## 1 Introduction

As human activity continues to degrade terrestrial ecosystems, the need for effective seed‐sourcing to restore productive and diverse plant communities is becoming more urgent (Miller et al. 2017), reinforcing calls for developing and supporting robust seed‐sourcing systems at local, national, and international levels (Prasse et al. 2010; PCA 2015; NNSP 2020; Cross et al. 2020; Erickson and Halford 2020; Pedrini and Dixon 2020; Hancock and Encinas‐Viso 2021; NASEM 2023). A primary consideration in plant community restoration is the geographic source, or provenance, of restoration seed (Hufford and Mazer 2003; Broadhurst et al. 2008; Breed et al. 2018). Plant populations are often adapted to conditions at sites where they have persisted for multiple generations and maladapted to sites with conditions dissimilar to the locations where they evolved (Linhart and Grant 1996; Leimu and Fischer 2008; Hereford 2009; Bucharova, Durka, et al. 2017; Baughman et al. 2019). Consequently, plants growing at their home sites, or sites with similar conditions to their home site, will commonly demonstrate higher fitness than plants from populations originating elsewhere and from different environments, a pattern known as local adaptation (Kawecki and Ebert 2004). Use of seed with adaptive characteristics matched to restoration site conditions can thus increase the probability of success in restoration seedings (Hamilton 2001; McKay et al. 2005; Bischoff et al. 2010; Johnson et al. 2010; Bucharova, Michalski, et al. 2017; Camarretta et al. 2020). As climate change shifts local environmental conditions away from historic norms, information on genetic and phenotypic trait variation of source populations will also be useful for matching seed sources to changing conditions at restoration sites (Vitt et al. 2022).

Common garden studies, in which plants from multiple source populations are grown together in one or more shared environments, allow the environmental component of trait variation to be controlled (Clausen et al. 1940; Kawecki and Ebert 2004). As a result, associations between population mean traits and the climates at their source locations primarily reflect genetically based differences among populations (Savolainen et al. 2007; Hereford 2009). These quantitative genetic trait‐climate associations are therefore informative about the distribution of adaptive genetic variation across a species' range, although they may also partially reflect neutral processes associated with demographic history (Leimu and Fischer 2008; Savolainen et al. 2013). Ideally, reciprocal transplant common gardens are used to test for local adaptation across a broad range of environments, including home sites (Kawecki and Ebert 2004; Wang et al. 2010; Blanquart et al. 2013; Johnson et al. 2022; Lortie and Hierro 2022). However, these experiments require considerable logistics and resources beyond what is generally available (see Wang et al. 2010 for a rare example). By contrast, genecological common garden, or provenance, studies grow populations together in one or a few controlled environments, providing an indirect but more logistically feasible estimate of climate‐associated adaptive genetic variation (Turesson 1923; Langlet 1971; Heslop‐Harrison 1964; Campbell 1974; St. Clair et al. 2005; Kilkenny 2015; Baughman et al. 2019). In addition to being indirect estimates of local adaptation, trait‐climate associations developed from genecological provenance studies provide a foundation for developing ‘empirical’ seed transfer guidelines for restoration species (e.g., St. Clair et al. 2013) and modeling maladaptation risk under climate change (Kilkenny 2015; Richardson and Chaney 2018).

Empirical seed transfer models are designed to help restoration practitioners make seed‐sourcing decisions informed by mapped trait‐climate associations (Ying and Yanchuk 2006; Kilkenny 2015; Edwards et al. 2019). Such models indicate which seed sources are most suitable for a given restoration site, or conversely, which restoration sites would be most appropriate for a given seed source. Models commonly take the form of fixed‐boundary seed zones, which simplify spatial variation by defining discrete geographic areas within which seed can be both sourced and used (e.g., Campbell 1986; St. Clair et al. 2013). Alternatively, focal‐point seed transfer models depict seed source suitability as a continuous gradient across a landscape, generated dynamically for a specific point of interest (i.e., restoration site or seed source location) without pre‐defined suitability thresholds (Rehfeldt 1984; Parker 1992; Parker and van Niejenhuis 1996). Both fixed‐boundary and focal‐point seed transfer maps can be generated through spatial projections of correlative models referred to as transfer functions, usually generated through multiple linear and polynomial regression. These functions can be used directly in developing focal‐point seed transfer models (e.g., Richardson and Chaney 2018). However, delineating fixed‐boundary seed zones requires additional, potentially subjective, steps for determining the placement of boundaries and the number of zones, considering the tradeoff between practicality (fewer, larger zones providing a broader pool of source populations for restoration seedings within a zone) and risk‐avoidance (more, smaller zones to reduce the risk of maladapted seed transfer) (Hamann et al. 2011; Kilkenny 2015). Given this tradeoff, restoration practitioners may benefit from more transparent and flexible fixed‐boundary seed zone delineations that can be adjusted by users to encompass broader or narrower transfer criteria, thereby providing scalability and accommodating various transfer risk tolerances and practical constraints.

An under‐utilized method for developing fixed‐boundary seed zones involves multivariate regression trees (Hamann et al. 2011; Liepe et al. 2016). Regression tree modeling offers a data‐driven approach for determining zone boundaries and an intuitive and explicit decision‐tree framework for understanding the relative importance of different predictor variables and their interactions (Breiman et al. 1984; De'Ath 2002; Hamann et al. 2011). Because of the hierarchical structure of regression tree models, the number of zones is not fixed but can vary depending on stopping criteria, which can be specified using complexity parameters, cross‐validation (Breiman et al. 1984; De'Ath 2002), statistically significant differentiation between nodes (Hothorn et al. 2006) or even post hoc biological or practical considerations, such as the trait plasticity of seed sources or their availability in relation to restoration needs. An additional advantage of regression trees is that they can incorporate categorical variables, such as subspecies identity or geographic region, and directly test their importance within the model (Hamann et al. 2011). Although regression trees are not ideal for modeling continuous transfer functions, and therefore focal‐point seed transfer models, ensemble variants of the regression tree algorithm, for example random forests (Breiman 2001) can be applied for this purpose.

Several additional evolutionary considerations can influence restoration seed‐sourcing decisions. Two of the most important are the role of genetic lineages in evolutionary patterns and processes and plasticity in trait expression across environments. Increasingly, demographic history and intraspecific genetic differentiation are being considered in the development of seed zones (Jørgensen et al. 2016; Massatti et al. 2018; Richardson et al. 2023). This information is mostly used to account for factors not directly related to local adaptation, such as the impact of seed transfer on the genetic integrity of local remnant populations and overall species genetic diversity (Massatti et al. 2020), but can also have adaptive consequences if distinct lineages have different trait‐climate associations (Shryock et al. 2021). High levels of trait plasticity within a species are often associated with persistence across a wide range of biotic and abiotic environments (Ghalambor et al. 2007; Chevin et al. 2010; Diamond and Martin 2021) and can be adaptive or facilitate future adaptation (Eshel and Metessi 1998; Pal and Miklos 1999; Kelly 2019) or, conversely, may slow or hamper adaptation (Price et al. 2003; Gibert et al. 2019). Therefore, the levels and patterns of trait plasticity can also be an important consideration in seed transfer guidance.

In this study, we characterize quantitative genetic variation of the grass *E. elymoides* (Raf.) Swezey (bottlebrush squirreltail), an important restoration species, across a core section of its range in the northern Great Basin and surrounding ecoregions (hereafter, Intermountain Region) in the western United States. Traits related to phenology, growth, reproduction, leaf morphology, and survival, measured in three common gardens, provide the basis for this characterization. We use associations between these traits and the climates at population source locations to develop empirical seed transfer guidelines, using a novel modeling framework that combines regression trees and random forests to produce both fixed‐boundary seed zones and focal‐point seed transfer models. We explicitly define and compare differences between our modeling approach and standard approaches to seed transfer guideline development, using the *E. elymoides* seed transfer models as a demonstration of our approach. We also provide analysis and guidance on how several species‐specific factors, discovered while conducting our analyses, might affect seed transfer guidance in *E. elymoides* ; specifically, subspecies differentiation in trait‐climate associations and plasticity in trait expression across common gardens. Lastly, we frame seed transfer guideline development as a whole, and our approach in particular, within the context of seed‐sourcing under global change.

## 2 Methods

### 2.1 Study Species

*Elymus elymoides* is a cool‐season, short‐lived, self‐pollinating, perennial bunchgrass native to semi‐arid regions of western North America (Jones 1998). It is found in a wide variety of habitats from the Pacific Coast to the Great Plains and from Canada to Mexico at elevations from sea level to 3500 m (Wilson 1963). *E. elymoides* is considered a “workhorse” species in native plant community restoration (Erickson 2008) and is one of the most commonly used species in the Intermountain Region (Jones 2019), where tens to hundreds of thousands of ha of post‐wildfire rehabilitation seedings are conducted each year (Pilliod et al. 2017). It can colonize disturbed sites (Hironaka and Tisdale 1963; Simmons and Rickard 2002), is relatively fire‐tolerant (Wright 1971; Young and Miller 1985; Ellsworth and Kauffman 2010) and is a potential competitor with the invasive grasses *Taeniatherum caput‐medusae* (L.) Nevski (medusahead) and *Bromus tectorum* L. (cheatgrass) (Clausnitzer et al. 1999; Booth et al. 2003; Humphrey and Schupp 2004; Leger 2008; Davies 2010; McGlone et al. 2012; Sheley and James 2014). Previous studies indicate that there is ecotypic divergence in *E. elymoides* , and at least five geographically overlapping subspecies have been identified in the region (Jones 1998; Jones et al. 2003; Larson et al. 2003). Trait variation has been documented in *E. elymoides* both between subspecies and at the population level (Clary 1975, 1979; Jones et al. 2003; Parsons, Jones, Larson, et al. 2011; Parsons, Jones, and Monaco 2011; Zhang et al. 2011; Atwater et al. 2015; Baughman et al. 2016; Solomon 2019; Blumenthal et al. 2021); and, this variation has been shown to be associated with source climates (Clary 1975; Blumenthal et al. 2021), and is potentially important in restoration outcomes (Leger et al. 2019, 2021).

### 2.2 Population Sampling and Experimental Design

**FIGURE 1:**
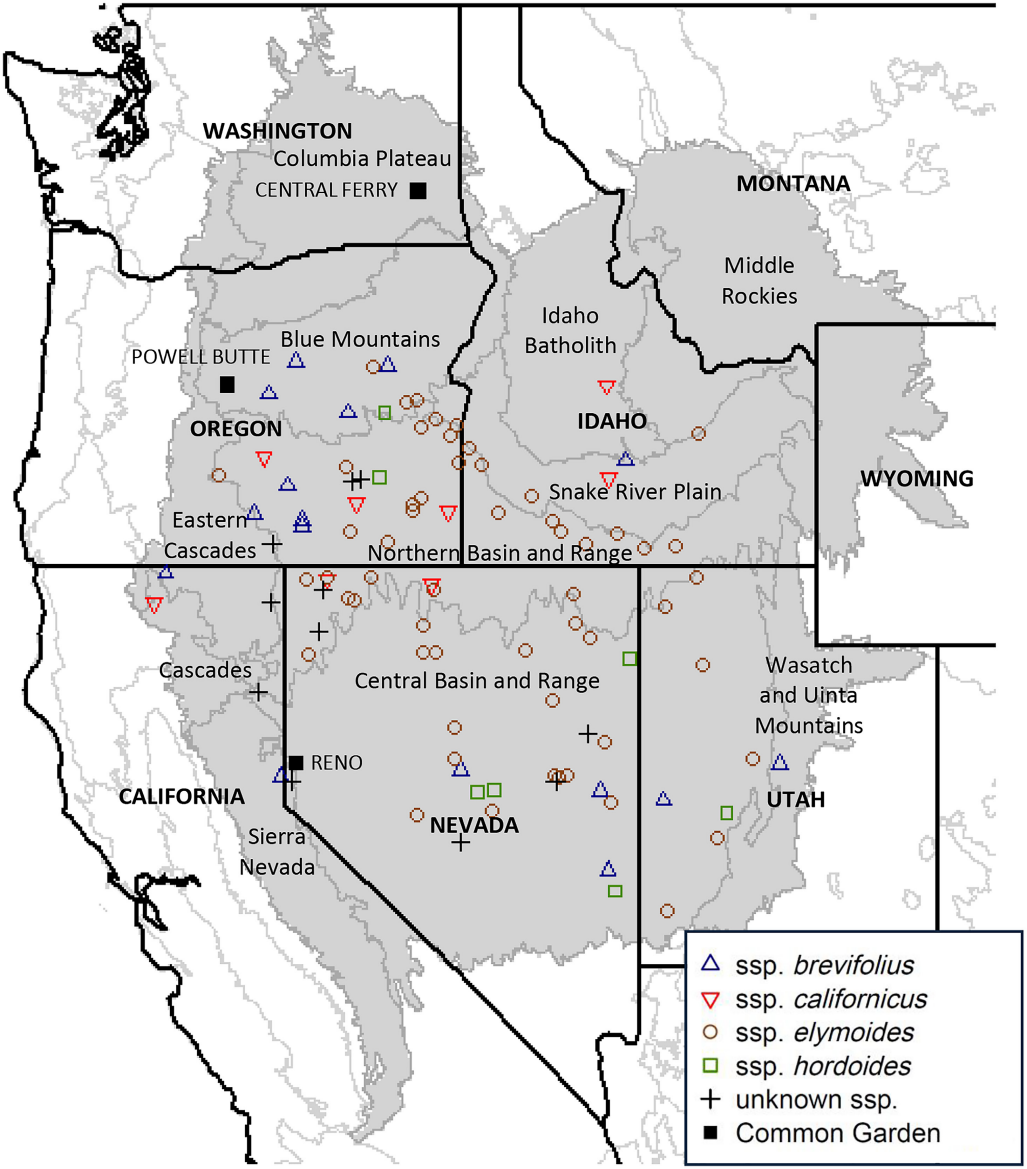
Source locations of *Elymus elymoides* populations (identified to subspecies, where possible) planted at three common gardens (Central Ferry, Powell Butte, Reno) in the Intermountain Region, western USA. Outlines of EPA Level III Ecoregions (Omernik and Griffith 2014) are shown, with focal ecoregions highlighted.

Seeds were collected by researchers and volunteers between 2007 and 2010 from 98 wildland *E. elymoides* populations located in the Intermountain Region across an area spanning five states and ten US Environmental Protection Agency (EPA) Level III Ecoregions (Omernik and Griffith 2014) (Figure 1). Seeds from two maternal families were collected from each population and put into cold storage until sown for germination. In 2011, we germinated seedlings in growth chambers, then transplanted them into Conetainers (Steuwe & Sons, Tangent OR) after 2 weeks and grew them in a greenhouse at Oregon State University for 18–41 weeks before planting, depending on when germination occurred. Due to low germination rates of many seed collections, especially older ones, several rounds of germination were needed for 88 populations to generate enough plants for out‐planting. Plants that germinated early were therefore kept under greenhouse conditions for longer than plants that germinated later. Plants were strategically clipped aboveground to equalize growth and development across populations. All plants were clipped at least once before out‐planting. Collection year and days in greenhouse were used to test for effects of pre‐planting conditions on trait expression in the common gardens (see Trait Analyses Section 2.5).

We transplanted juvenile plants to common gardens in fall 2011 at three test sites: Central Ferry, Washington; Powell Butte, Oregon; and Reno, Nevada (Figure 1). One individual per maternal family was randomly assigned to each of six blocks in each garden (i.e., six individuals per maternal family per garden totaling twelve individuals per population). Blocks were planted with 100 cm spacing between rows and 80 cm between plants within rows. A set of 1176 test plants was installed in each garden for a total of 3528 plants across all gardens. To minimize edge effects, a single buffer of non‐measured plants was planted along each edge of each garden at the same spacing as the measured plants. Buffer plants were randomly selected from populations with excess individuals.

### 2.3 Trait Measurements

Traits were measured at common gardens in 2012 and 2013; however, only the 2013 data were analyzed to allow for a year of growth and acclimatization to further minimize potential germination, grow‐out, and transplant effects. Measured traits assessed plant growth, leaf morphology, phenology, reproduction, and survival (Table 1). Phenology was measured at least weekly across the season, and at least every other day when significant phenological stage transitions were occurring. Traits related to growth, leaf morphology, and reproduction were measured near the end of the growing season after seed maturation and before fall senescence (late July or August, depending on the garden). Above‐ground biomass for every living plant was harvested, then dried and weighed at the end of the growing season in 2013. Survival was measured for the interval between the beginning of the 2012 and end of the 2013 growing seasons.

**TABLE 1:** Traits measured on *Elymus elymoides* plants grown at three common gardens.

Trait type	Trait	Description
Growth	Biomass	Total above ground biomass at the end of the growing season
	Plant height	Height off the ground at the highest point of self‐supported vegetation occurrence; taken at or after the peak of the growing season prior to any occurrence of senescence
Reproduction	Inflorescence number	A count or estimate of the total number of inflorescences present at the end of the growing season; full counts were made on plants with up to ~40–50 inflorescences; for plants with higher inflorescence numbers, counts were made on roughly half or a quarter of the inflorescences present and then multiplied appropriately to derive a count estimate
Leaf morphology	Leaf length	Length of representative flag leaf; taken near or after the peak of the growing season, before any occurrence of senescence
	Leaf width	Width of representative flag leaf at the widest point; taken near or after the peak of the growing season before any occurrence of senescence
	Leaf ratio	Ratio of leaf length to leaf width
Phenology	Heading date	Date of first observation of anthesis
	Maturation date	Date before the first observation of seed dispersal
Survival	Survival	Survival of transplants from the second to the third year

### 2.4 Climate Data

Climate data were obtained from ClimateWNA version 5.40, an application with built‐in functions for interpolating historic weather station data and constructing time‐specific climate variables for locations in western North America (Wang et al. 2012, 2016). We extracted 30‐year (1981–2010) normals for 23 annual climate variables (Table S1) at the population source locations, using a 0.057 × 0.057 decimal degree (ca. 6 km × 6 km) grid. Annual climate variables were also extracted at the common garden locations for the 3 years (2011, 2012, 2013) during which plants were outplanted and grown for the purpose of understanding conditions that plants were exposed to during the course of the study. Unlike climate data for source locations, climate information for gardens was not directly used in trait‐climate analyses and seed transfer modeling.

### 2.5 Trait Analyses

For each trait, we estimated the proportion of variance attributable to common garden, block (nested within garden), population, and family (nested within population), treating all factors as random effects in a variance components model implemented in *lme4* in R (Bates et al. 2015; R Core Team 2016). Traits measured as counts (inflorescence number) or binary numbers (survival) were modeled using the Poisson (log link) and binomial (logit link) error distributions, respectively, while other variables were modeled using the Gaussian distribution with the identity link. Residual error variance components were not automatically generated in *lme4* for models using non‐Gaussian error distributions, but were estimated using the sigma function in R. Most trait variation occurred at the garden level, consistent with substantial phenotypic plasticity in plants. To characterize this trait plasticity at the population level, we conducted a second set of variance components analyses separately for each garden, again using only random effects. Population‐level trait plasticity was quantified for each trait as the standard deviation of population mean trait values across the three gardens. We then estimated Pearson correlations between population‐level trait plasticity and climatic conditions at population source locations. The resulting correlation coefficients were used to identify traits for which population‐level variation in trait plasticity was associated with climatic conditions at source locations.

We also used correlation analyses to examine potential effects of seed collection year and days in greenhouse on subsequent trait expression of transplants at common gardens. We found instances of significant correlations for some of these traits (biomass, height, leaf width, heading date, maturation date, survival) at one or more gardens (Table S2). These correlations were negative in all cases except for survival, which was positively correlated with days in the greenhouse at the Reno garden (Table S2). However, after accounting for the covarying effects of climate variables using semi‐partial correlations, none of the associations between collection year or days in greenhouse and the trait variables remained significant. We concluded that collection year and days in greenhouse affected measured traits, but that these effects were of minor importance for our trait‐climate analyses.

### 2.6 Seed Transfer Modeling

**FIGURE 2:**
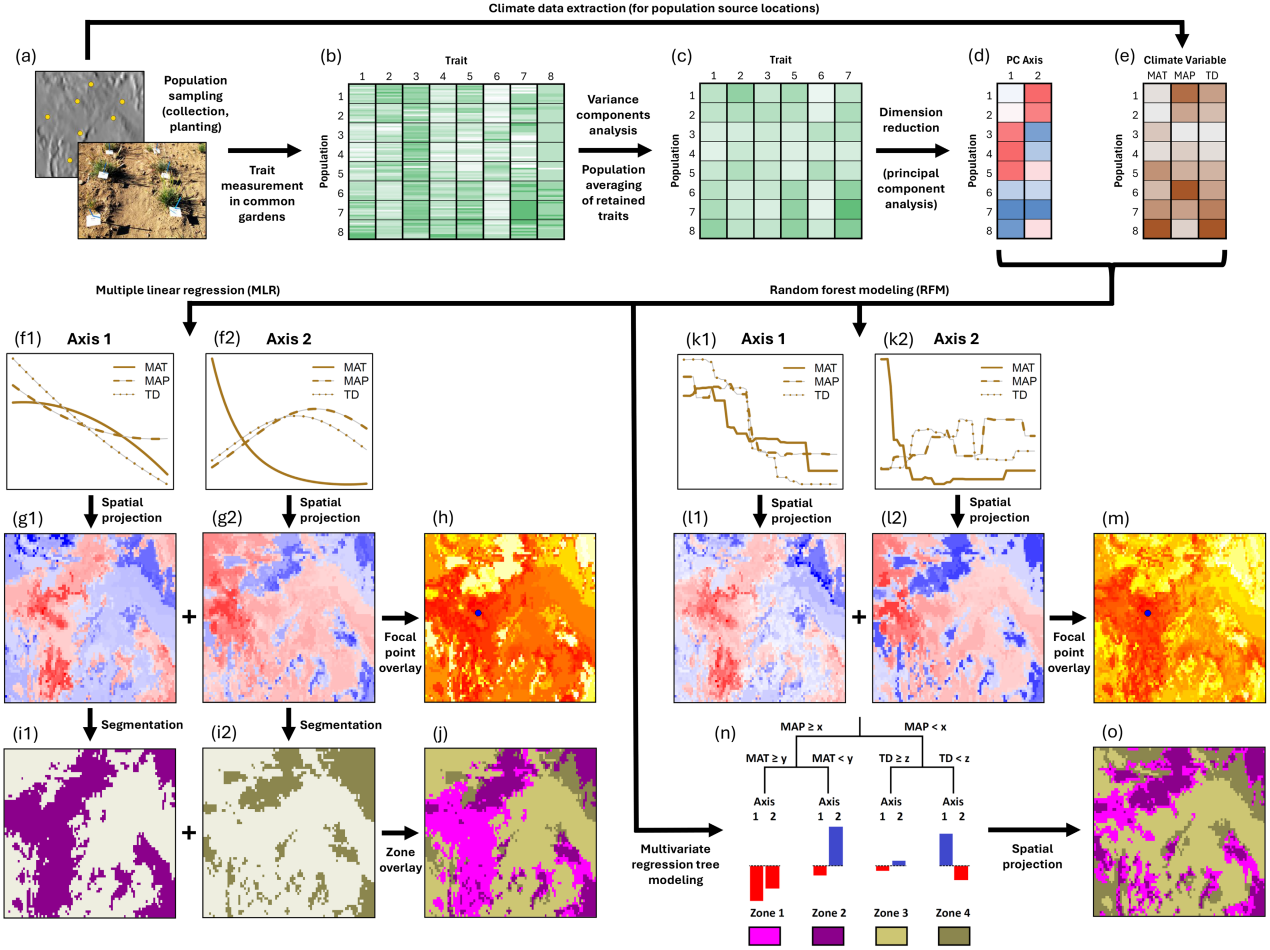
Schematic illustration of methodological approaches for developing seed transfer models and maps from trait and climate data. Top row (a–e) shows initial steps shared by two approaches, shown in parallel: (1) the approach commonly used in previous studies (lower left, f‐j) and (2) the approach followed in the current study (lower right, k–o). (a) example locations of populations where seed is collected and plants growing in a common garden; (b) individual trait values measured for plants in common gardens; (c) population means for traits with population‐level variation, as determined by variance components analysis; (d) composite trait axes obtained from a dimension reduction technique such as principal component analysis; (e) values of climate variables at population source locations, here represented by mean annual temperature (MAT), mean annual precipitation (MAP) and seasonal temperature difference (TD); (f) correlative relationships between composite trait axes (*x*‐axis) and climate variables (*y*‐axis) obtained through multiple linear regression (MLR); (g) spatial projections of MLR models onto mapping region; (h) focal‐point seed transfer models from overlaying MLR spatial projections; (i) zone divisions from segment partitioning of MLR spatial projections; (j) fixed‐boundary seed zones from overlay of zone divisions; (k) correlative relationships between composite trait axes (*x*‐axis) and climate variables (*y*‐axis) obtained through random forest modeling (RFM); (l) spatial projections of RFM models onto mapping region; (m) focal‐point seed transfer models from overlaying RFM spatial projections; (n) multivariate regression tree model predicting composite trait axes from climate variables; (o) fixed‐boundary seed zones from spatial projection of multivariate regression tree model.

Fixed‐boundary seed zones and focal‐point seed transfer models were developed using modifications of a methodology (Figure 2) that has been widely applied for genecological provenance studies of trees and grasses in western North America (e.g., St. Clair et al. 2005, 2013; Johnson et al. 2012, 2015, 2017; Johnson and Vance‐Borland 2016). This methodology builds seed transfer models from trait data measured at one or more common gardens by the following steps: (1) identify traits exhibiting variation among populations (see Trait Analyses Section 2.5), (2) convert population trait means into a set of composite trait axes using a dimension reduction technique such as principal component analysis (PCA) or canonical correlation analysis (CCA), (3) fit correlative models (transfer functions) linking trait axes to climatic conditions at population source locations, (4) generate maps of predicted values for each trait axis (the basis of focal‐point seed transfer models) by projecting their correlative models onto a geographical region of interest, and (5) delineate seed zones by partitioning trait axes into segments and combining their projections as spatial overlays (Figure 2a–j). We followed this methodology with two exceptions. In step 3, correlations between trait axes and climate variables were estimated using random forest models rather than the multiple linear or polynomial regressions used in most previous studies. In step 5, multivariate regression trees were applied as an alternative to the axis segment partitioning approach (Figure 2k–o).

Traits exhibiting variation among populations (from variance components analysis; see Trait Analyses Section 2.5) were integrated into a data matrix containing mean trait values for each population (Figure 2a–c). Because of high variation in trait expression between common gardens, we treated each trait × garden combination as a separate trait data column. The trait × garden data matrix was decomposed into a set of uncorrelated axes (Figure 2d) through principal component analysis (PCA) using the *ade4* R package (Dray and Dufour 2007; R Core Team 2016), with the matrix centered and scaled to standardize across different units of measurement. The number of significant principal component (PC) axes was determined using a Monte Carlo test on the sum of eigenvalues (Dray and Dufour 2007; Dray 2008).

Relationships between trait variation, represented by the matrix of significant PC trait axes, and climates of population source locations (Table S1, Figure 2e) were modeled using multivariate regression tree modeling to define fixed‐boundary seed zones (Figure 2n,o). Multivariate regression tree modeling recursively partitions a multivariate response variable by identifying, at each partition, the predictor variable that minimizes the sum of squared distances of centroids within groups while maximizing them between groups (De'Ath 2002). For our regression tree models, the response variable was the set of significant PC axes and the predictors were climate variables at source locations (Table S1). To examine whether responses differed among subspecies, a secondary regression tree model was developed, which included subspecies as a predictor variable in addition to climate variables. Multivariate regression tree modeling was implemented in the *mvpart* R package (R Core Team 2016) with the requirement that each tree partition contained at least 5 data points (populations). Tree models were grown to maximum size at a complexity parameter of 0.015, meaning that each partition included in the model was required to reduce residual sum of squared distances by at least 1.5% (Therneau et al. 2023). These settings ensured that partitions were balanced and led to a reasonable number of interpretable seed zones, within the practical range of 11–15 zones established by previous studies for other grass species at similar scales in the Intermountain region (St. Clair et al. 2013; Johnson et al. 2012, 2015, 2017; Johnson and Vance‐Borland 2016).

We used random forest modeling to develop focal‐point seed transfer models, building separate models for each PC trait axis (Figure 2k–m). Random forest models can be used to characterize the correlative relationship between a set of predictors and a univariate response using an ensemble of regression trees generated with random subsampling of eligible predictors at each tree partition (Breiman 2001; Jeong et al. 2016; Zanella et al. 2017). Using the same set of climate predictors as the regression tree models (Table S1), we built random forest models characterizing the relationship between climates at population source locations and PC axis values. Modeling was carried out for each significant PC axis using the *randomForest* R package with default settings (an ensemble of 500 trees, subsampling one‐third of the predictor variables at each split, sampling cases with replacement) (Liaw and Wiener 2002; R Core Team 2016). Variable importance was determined for each predictor variable of each random forest model as the average decrease in residual sum of squares (Liaw and Wiener 2002). We used partial dependence plots (Friedman 2001; Liaw and Wiener 2002) to evaluate the marginal effects of the most important predictor variables. Each random forest model was spatially projected onto the mapping region to visualize the spatial distributions of values for each PC axis, in addition to using the combined model projections as a focal‐point seed transfer function, in which seed transfer suitability for a given point on the landscape is measured by multivariate Euclidean distance in PC trait space (Teng et al. 2020). Locations closer to each other in trait space, defined by orthogonal PC axes, were inferred to have greater suitability for seed transfer.

We projected multivariate regression tree and random forest models geographically beyond population source locations (Figure 2l,o) using the *raster* R package (Hijmans 2015; R Core Team 2016) on climate layers extracted from ClimateWNA. The mapping area consisted of the EPA Level III Ecoregions (Omernik and Griffith 2014) where one or more populations had been collected, plus the climatically similar Columbia Plateau ecoregion where the Central Ferry common garden is located (Figure 1). We included the California portion of the Cascades ecoregion, but not the spatially separate Oregon/Washington portion, which we did not sample (Figure 1). Within these ecoregions, we masked areas of model uncertainty lying beyond the climatic limits of source locations; specifically, areas that were one standard deviation beyond the sampled range of any of the 23 climate variables (Table S1).

## 3 Results

### 3.1 Trait Variation Among Gardens and Populations

Measured trait values differed across the three common gardens (Table 2), reflecting the expected trait plasticity in response to garden conditions. Plants were larger, produced more inflorescences, and matured later at Powell Butte compared to the other two gardens (Table 2). Leaves were the largest (in both length and width) at Central Ferry (Table 2). Plants at Reno had much lower biomass and produced fewer inflorescences than elsewhere, although they were similar in height to plants at Central Ferry (Table 2). Survival was highest at Powell Butte and lowest at Reno (Table 2). These differences in trait expression likely resulted from different conditions at each garden. During the period of the study, total precipitation was highest at Central Ferry, although summer precipitation and precipitation as snow were highest at Powell Butte, where temperatures were lowest (Table S3). Reno had the lowest precipitation and the greatest seasonal variation in temperature during the study period (Table S3).

**TABLE 2:** Mean trait values of 98 *Elymus elymoides* populations measured at three common gardens.

	Central ferry		Powell butte		Reno	
	Mean	SD	Mean	SD	Mean	SD
Biomass (g)	33.6	(22.7)	63.8	(27.9)	3.3	(1.7)
Plant height (cm)	18	(2)	36	(7)	16	(4)
Inflorescence number	103	(37)	209	(68)	13	(6)
Leaf length (cm)	14.1	(1.5)	7.5	(1.2)	7.4	(1.1)
Leaf width (cm)	0.44	(0.07)	0.37	(0.06)	0.27	(0.05)
Leaf ratio	34.4	(6.7)	21.1	(4.1)	31.1	(7.3)
Heading date	131	(5)	144	(6)	141	(7)
Maturation date	163	(2)	180	(5)	160	(8)
Survival (%)	92	(10)	97	(7)	74	(18)

Common garden and population were major contributors to variation for most measured traits (Table 3; Figure S1). Most of the explained trait variation was due to garden effects, ranging from 23% for leaf ratio to 72% for plant height, whereas variation explained by population ranged from 3% for leaf length and inflorescence number to 24% for heading date (Table 3). Block never explained more than 6% and family never more than 7% of the variation in any trait (Table 3). Residual unexplained variation ranged from 17% for plant height to 64% for leaf ratio (Table 3). Patterns of explained variation varied among gardens when variance components were analyzed on a garden‐by‐garden basis (Figure S1). Most traits had lower population‐level variation at Reno than Central Ferry and Powell Butte, although phenology traits (heading and maturation dates) and survival were outliers in this regard (Figure S1). Leaf ratio had negligible (< 0.001%) population‐level variation at Reno, and because of this, we opted to exclude it from subsequent analyses. The remaining 26 trait × garden combinations (from 9 traits and 3 gardens) were used as input variables for PCA.

**TABLE 3:** Results of variance components analysis for traits of 98 *Elymus elymoides* populations measured at three common gardens.

Trait	Garden	Block	Population	Family	Residual
Biomass	49.0	1.0	16.4	1.9	31.7
Plant height	72.3	2.0	6.2	2.2	17.3
Inflorescence number	54.8	1.0	3.0	2.4	38.9
Leaf length	68.2	0.3	2.8	1.9	26.8
Leaf width	37.5	1.6	10.9	3.0	47.0
Leaf ratio	23.1	1.0	7.0	4.8	64.1
Heading date	39.4	1.1	24.5	4.8	30.2
Maturation date	69.6	1.3	7.3	1.4	20.4
Survival	37.0	5.7	13.2	6.6	37.6

*Note:* Values shown are decimal percent variance attributed to garden, block within garden, population, family within population, and residual.

Of the 26 PC trait axes resulting from PCA, 6 were significant, and these 6 axes collectively accounted for 77% of the total variance. Loadings of trait × garden variables on PC axes revealed patterns of contrasting trait expression (Table S4). On the first PC axis, traits with negative loadings included heading date and maturation date at all gardens; and biomass, plant height, and inflorescence number at Central Ferry and Powell Butte; contrasting with positive loadings for survival at Reno and leaf ratio at other gardens (Table S4). The second PC axis showed a contrast between leaf ratio (high loadings) and leaf width (low loadings) across gardens (Table S4). The third PC axis was strongly related to traits measured at Reno, separating biomass, plant height, leaf length, and inflorescence number (negative loadings) from heading date, maturation date, and survival (positive loadings), along with positive loadings for inflorescence number at the other gardens (Table S4). The fourth PC axis separated leaf length, leaf ratio, heading date, and maturation date (positive loadings) from the remaining variables (Table S4). The fifth and sixth PC axes captured additional contrasts among various trait × garden combinations (Table S4).

Variation in population‐level trait expression across common gardens, indicative of plasticity, was significantly correlated with climate at population source locations in multiple cases where this relationship was tested (Table 4). Relationships between trait plasticity and climate were also evident in patterns of trait variation among climate‐based seed transfer zones (Figure S2). Plasticity of biomass, plant height, leaf width, and survival was positively correlated with one or more precipitation variables and was generally negatively correlated with variables indicating warmer temperatures, longer frost‐free periods, or greater aridity (while variables where higher values indicate cooler temperature or frost regimes, that is chilling degree‐days [DD_0], heating degree days [DD_18], and beginning date for the frost‐free period [bFFP], were negatively correlated) (Table 4). A similar pattern, but with fewer significant correlations, emerged for inflorescence, leaf length, and heading date traits (Table 4). Maturation trait plasticity followed an opposite pattern of negative correlations with precipitation and positive correlations with most temperature, frost, and aridity variables (Table 4). No significant correlations were detected for leaf ratio plasticity.

**TABLE 4:** Correlations between trait plasticity and climates at population source locations for 98 populations of *E. elymoides* grown at three common gardens.

	Biomass	Plant height	Inflorescence number	Leaf length	Leaf width	Heading date	Maturation date	Survival
**Precipitation**								
MAP	0.36	—	—	—	0.32	0.26	—	0.50
MSP	0.28	0.34	—	—	0.25	—	−0.23	0.31
PAS	0.24	—	—	—	—	—	—	0.47
RH	—	—	—	—	—	—	—	0.32
**Temperature**								
EMT	—	−0.25	—	—	—	—	0.31	—
EXT	−0.30	−0.39	−0.23	—	−0.28	—	0.39	−0.32
MAT	−0.32	−0.42	−0.31	−0.26	−0.32	—	0.47	−0.31
MCMT	—	—	—	—	—	—	—	−0.24
MWMT	−0.48	−0.48	−0.39	−0.31	−0.41	—	0.51	−0.29
TD	−0.51	−0.4	−0.36	−0.30	−0.37	—	0.40	—
**Degree‐days**								
DD_0	—	0.25	—	—	—	—	−0.29	0.30
DD_18	0.28	0.39	0.29	0.23	0.29	—	−0.45	0.32
DD5	−0.40	−0.46	−0.36	−0.3	−0.36	—	0.51	−0.28
DD18	−0.44	−0.5	−0.38	−0.32	−0.41	—	0.51	−0.23
**Frost**								
NFFD	—	−0.45	−0.32	−0.25	−0.26	—	0.54	—
FFP	−0.31	−0.47	−0.38	−0.29	−0.31	—	0.56	—
bFFP	0.32	0.51	0.36	0.27	0.31	—	−0.58	—
eFFP	−0.26	−0.35	−0.36	−0.27	−0.26	—	0.48	—
**Aridity**								
AHM	−0.43	−0.44	—	—	−0.48	−0.25	0.38	−0.37
SHM	−0.33	−0.45	−0.22	—	−0.31	—	0.41	−0.28
CMD	−0.37	−0.34	—	—	−0.33	—	0.29	−0.42
Eref	−0.31	−0.25	−0.23	—	−0.24	—	0.27	−0.40

*Note:* Plasticity was measured as the standard deviation of mean measured trait values across gardens. Climates at source locations were acquired from ClimateWNA (Wang et al. 2016) as 30‐year normals (1981–2010) for 23 variables (see Table S1). Pearson correlation coefficients are shown if statistically significant (*p* < 0.05), with *p* values adjusted for multiple tests using the false discovery rate (Benjamini and Hochberg 1995). Leaf ratio and MAR are omitted from columns and rows, respectively, due to lack of significant correlations.

### 3.2 Fixed‐Boundary Seed Zones Derived From Multivariate Regression Trees

**FIGURE 3:**
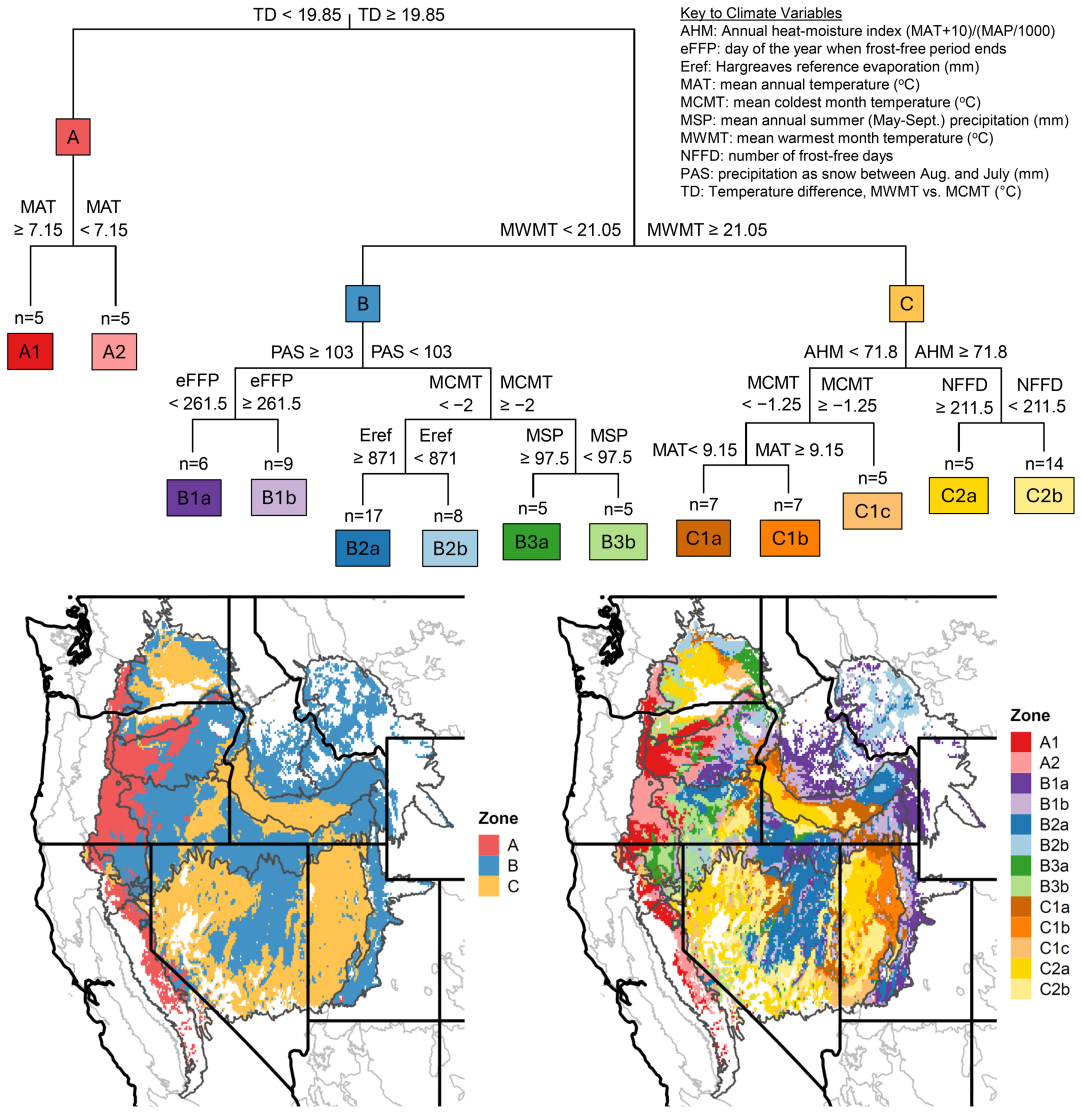
Fixed‐boundary seed transfer zones for *Elymus elymoides* derived from multivariate regression trees. Upper part of the figure shows the decision tree of the multivariate regression tree model, associating trait PC trait axes (Table S4) with climate (Table S1) at population locations, from which the seed zones were derived. Colors and code names at terminal nodes of the regression tree model correspond to the mapped seed zones; n indicates the number of populations represented in each terminal node. Lower part of the figure shows the spatial projection of these seed zones across the Intermountain Region of western North America at two resolutions (3 internal nodes on the left; 13 terminal nodes on the right). Mapped area is defined by EPA Level III Ecoregions (Omernik and Griffith 2014; Figure 1), omitting areas beyond 1 standard deviation of the sampled climate range.

Multivariate regression tree modeling using population source location climate variables as predictors of multivariate trait variation resulted in a tree model with 13 terminal nodes, each represented by 5–17 populations (Figure 3). When projected onto the mapping region, this model defined seed zones that we grouped at three levels of the tree hierarchy (Figure 3). The first level contained three primary zones (A, B, C) differentiated by seasonal temperature difference (TD) and mean warmest month temperature (MWMT) at the first two partitions of the tree (Figure 3). Each primary zone was further partitioned into 2–3 secondary zones (1, 2, 3) and up to three tertiary zones (a, b, c) differentiated by a variety of precipitation, temperature, frost, and other climate variables (Figure 3, Table S1). Partitions defining the primary, secondary, and tertiary zones accounted for 22%, 14%, and 12% of the variance of the model, respectively, with 52% remaining as error variance.

Zones occupied distinct geographic areas and elevational bands within the mapping region (Figure 3). The A zones, distinguished from other zones by having lower seasonal temperature difference, were located primarily on the western side of the region in the vicinity of the Sierra Nevada, Cascades, and western flank of the Blue Mountains (Figure 3). Within this area, A2 occupied higher elevation, cooler, and wetter climates than A1 (Figure 3). The B3 zones were also concentrated on the western side of the region in areas with relatively low seasonal temperature difference and relatively high minimum winter temperatures, though not as high as nearby A1 (Figure 3). Farther east, zones B1, B2, C1, and C2 were positioned sequentially along a gradient from cooler, snowier climates at higher elevations of interior mountain ranges to warmer, more arid climates at lower elevations (Figure 3). Further subdivisions of the B and C zones were aligned with elevation or other geographic features associated with contrasting temperature, precipitation, and aridity (Figure 3). Portions of some zones, especially at the highest and lowest elevations of the region, occurred in climates beyond the range sampled by the study and were therefore mapped as areas of uncertainty (Figure 3).

Mean trait values of populations originating from each zone provided a basis for interpreting trait‐climate associations (Figures S3 and S4). Patterns of trait expression differed at each common garden, but in general, plants from milder climates (lower seasonal temperature difference, cooler, or less arid) were larger, produced more inflorescences, and had wider leaves and later phenology than those from harsher climates (Figures S3 and S4). Leaves were widest in populations from higher elevation areas in the western and central part of the region (zone A2), and narrowest from lower elevation areas (zone C2) (Figure S4). Leaf ratio exhibited a pattern similar to leaf width, but in the opposite direction, with high values in populations from warmer and drier climates at lower elevations (Figures S3 and S4). The earliest heading and maturation dates were observed in populations from the lowest and driest areas (zone C) (Figures S3 and S4). Biomass, plant height, and inflorescence number also tended to be lowest in populations from relatively arid areas, except at Reno where plants from some of the most arid zones (C1c, C2b) were relatively large and produced more inflorescences than plants from adjoining zones (Figure S4). Survival tended to be lower in plants from more arid zones, although plants from certain zones that were marginally less arid (C1b, C2b) had relatively high survival (Figure S4).

### 3.3 Focal‐Point Seed Transfer Models Derived From Random Forests

**FIGURE 4:**
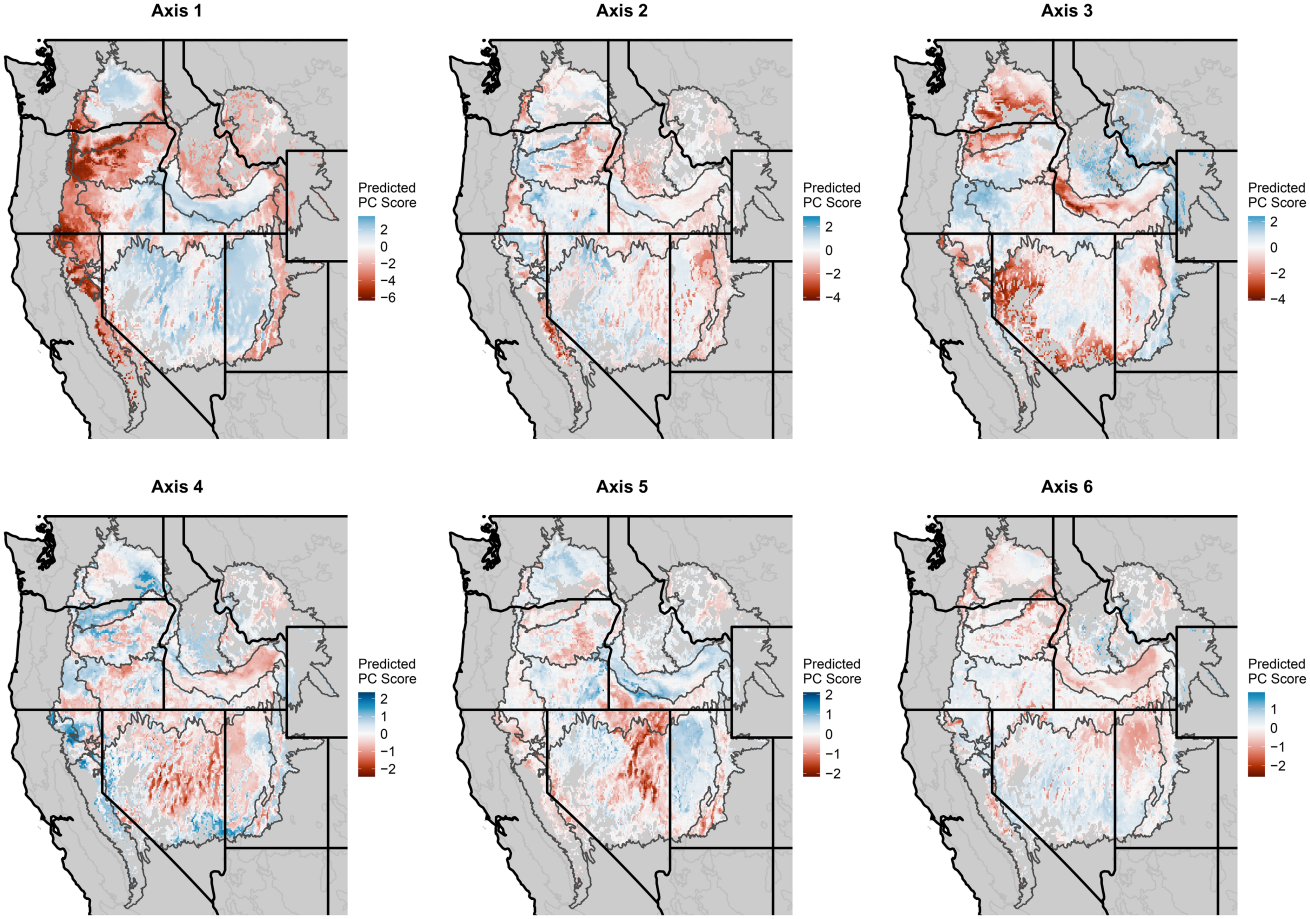
Spatial projections of transfer functions for *Elymus elymoides* modeled using random forests for six principal component axes of traits measured at three common gardens. Ecoregions of the western USA are shown (see Figure 1).

**FIGURE 5:**
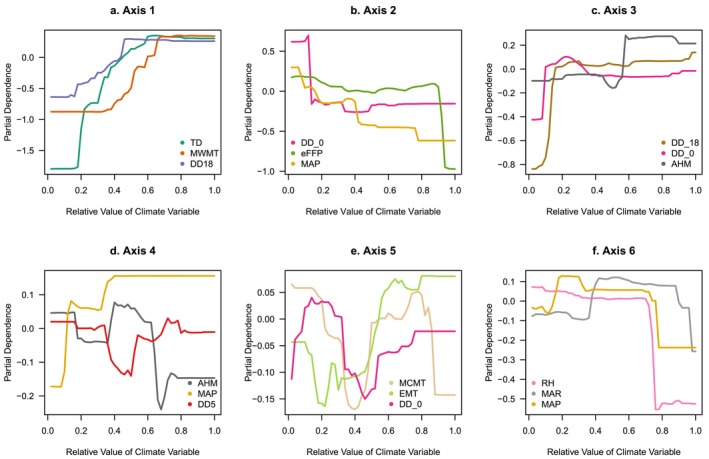
Partial dependence plots from random forest models showing the marginal effects of the three most important climatic predictors. Six random forest models were fitted separately, each using one of the six trait axes—derived from principal component analysis of traits measured in three common gardens—as the response variable. AHM, annual heat moisture index; DD_0, degree‐days below 0°C; DD_18, degree‐days below 18°C; DD18, degree‐days above 18°C; DD5, degree‐days above 5°C; eFFP, day of the year when frost‐free period ends; EMT, extreme minimum temperature over 30 years; MAP, mean annual precipitation; MAR, mean annual solar radiation; MCMT, mean coldest month temperature; MWMT, mean warmest month temperature; RH, mean annual relative humidity; TD, temperature difference between MWMT and MCMT.

Transfer functions derived from random forest models uncovered patterns of trait variation that were expressed as continuous fields. Each modeled PC trait axis had a unique spatial distribution reflecting its relationship with climate variables within the mapping region (Figure 4). Importance values varied across PC axes and predictor variables (Table S5). Partial dependence plots showed that the most important climatic variables generally increased or decreased along the main PC axes, although not necessarily uniformly or smoothly (Figure 5). Important climatic variables included seasonal temperature difference, mean warmest month temperature, and cooling degree days (DD18) for Axis 1 (Figure 5a); chilling degree days, frost‐free period ending date (eFFP), and mean annual precipitation (MAP) for Axis 2 (Figure 5b) and variables related to temperature, precipitation, aridity, and radiation for the remaining PC axes (Figure 5c–f).

**FIGURE 6:**
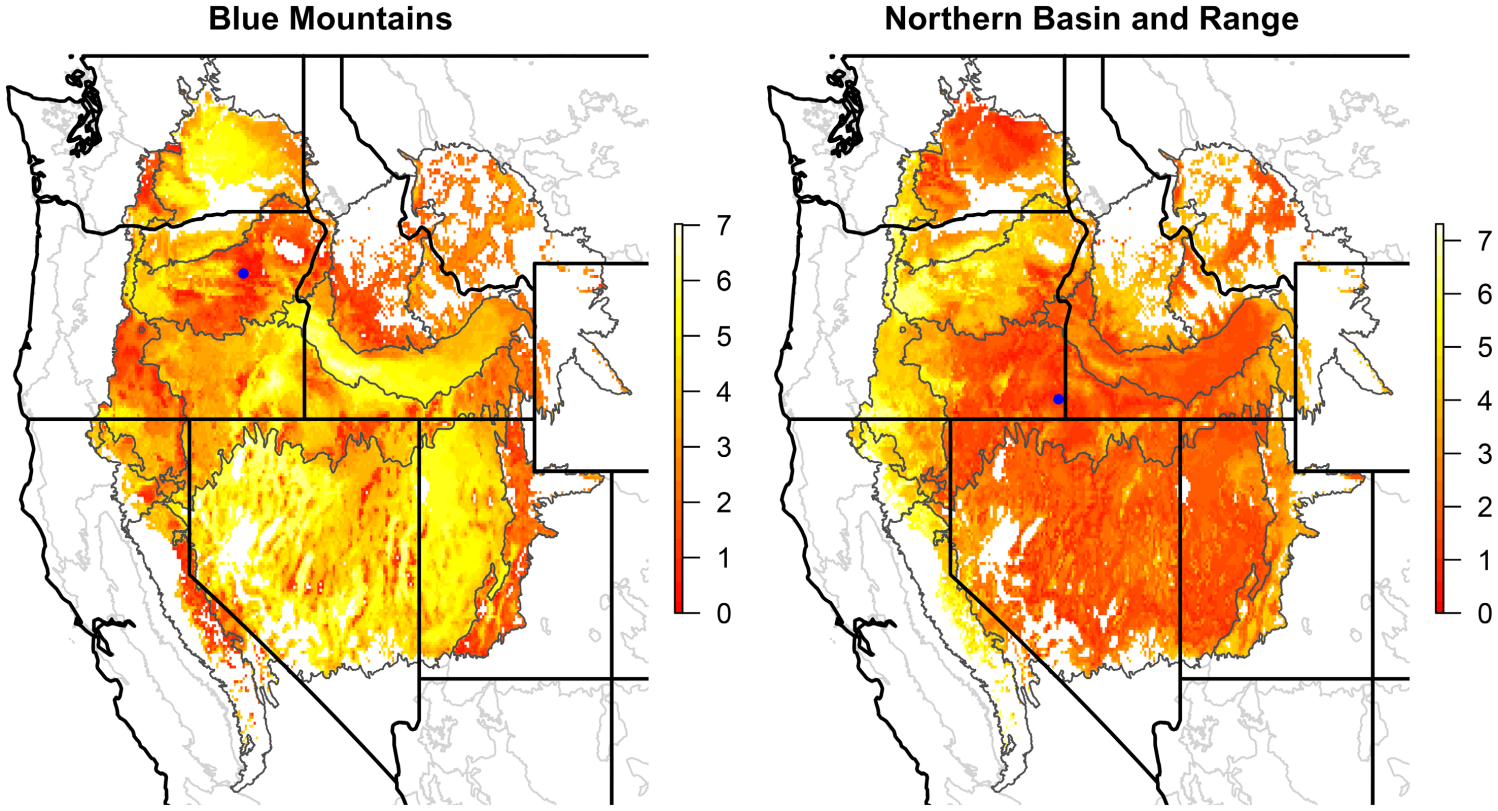
Examples of focal point seed transfer models developed from transfer functions of *Elymus elymoides* , mapped across ecoregions of the Intermountain Region, western USA (see Figure 1). Selected focal points (blue dots) are at centroids of the Blue Mountains ecoregion (left) and the Northern Basin and Range ecoregion (right). Redder coloring indicates lower Euclidean distance from focal points along six principal component axes of traits measured at three common gardens, projected spatially using relationships with climate variables.

Figure 6 illustrates an application of the transfer functions to predict the degree to which traits of populations throughout the mapping region are likely to resemble those at selected focal points, that is a focal‐point seed transfer model (Rehfeldt 1984; Parker 1992; Parker and van Niejenhuis 1996). Overall trait dissimilarity between points in geographic space is calculated as the predicted multidimensional Euclidean distance between their respective positions in PC trait space. Populations that are spatially proximate to focal points are likely to have similar traits, but spatially distant points may also have high predicted trait similarity if they are climatically similar according to the transfer function (Figure 6).

### 3.4 Seed Transfer Zones Incorporating Subspecies Identity

Four subspecies of *E. elymoides* were identified among the populations grown in the common gardens: ssp. *elymoides* (57 populations), ssp. *brevifolius* (16 populations), ssp. *californicus* (9 populations), and ssp. *hordeoides* (8 populations) (Figure 1). The ssp. *brevifolius* populations were putatively assigned to “Group C”, a genetically distinct ecotype that is described as occurring at mid to upper elevations in southern Idaho (Jones et al. 2003; Larson et al. 2003). However, Group C is nearly morphologically indistinguishable from other ssp. *brevifolius* groups, and the geographic distributions of these groups overlap. Additionally, several populations identified as Group C in this study were collected outside of the range where it is believed to occur. Therefore, ssp. *brevifolius* groups are not distinguished in our analysis. There were also 11 *E. elymoides* populations that could not be identified to subspecies (Figure 1). For analysis, these populations were labeled as unique taxa so that they could segregate independently in partitions of the regression tree.

**FIGURE 7:**
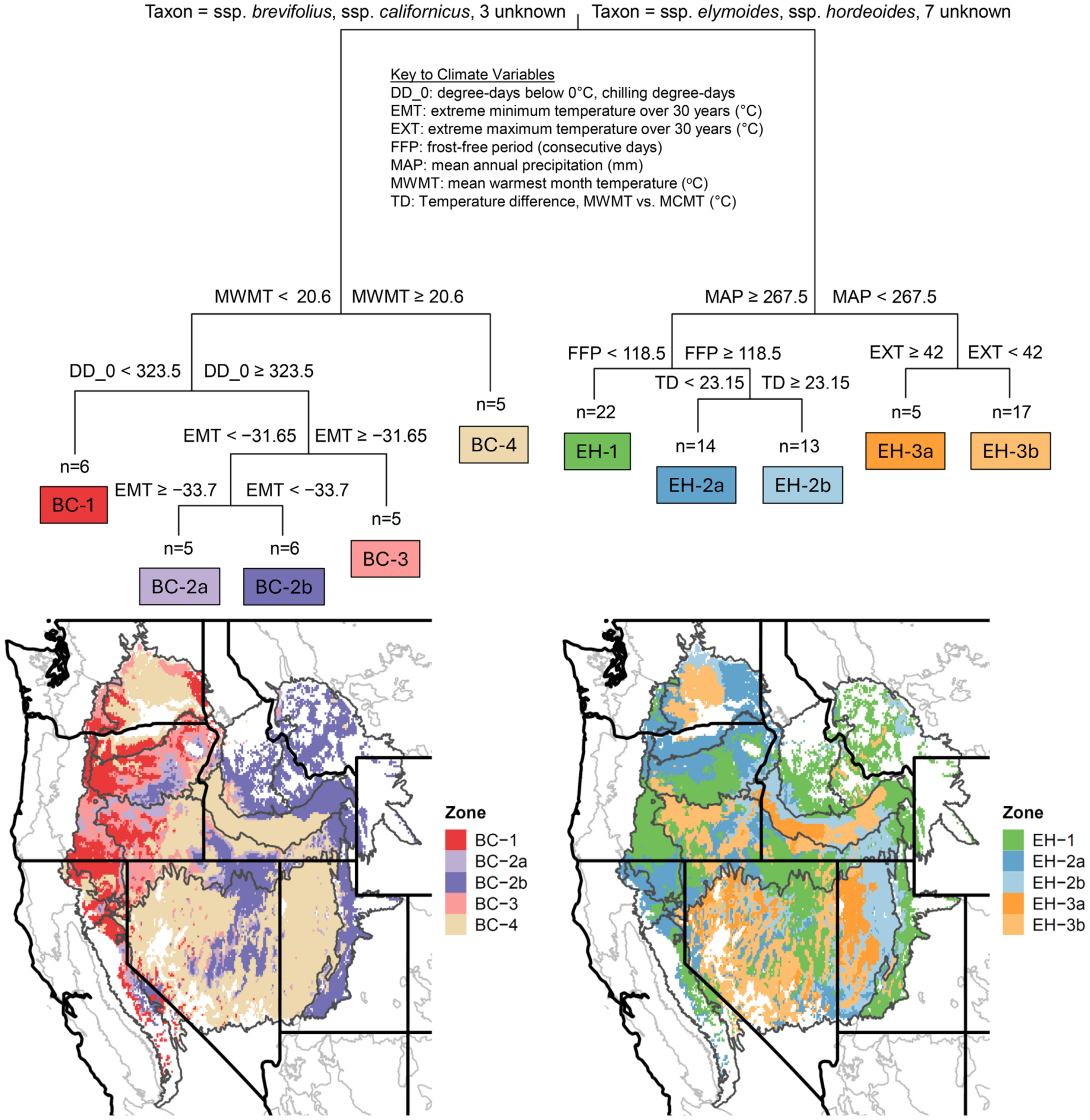
Fixed‐boundary seed transfer zones for *Elymus elymoides* subspecies groups derived from multivariate regression trees. Upper part of the figure shows the decision tree of the multivariate regression tree model, associating trait PC trait axes (Table S4) with climate (Table S1) at population locations with taxonomic status as an additional predictor variable, from which the seed zones were derived. Colors and code names at terminal nodes correspond to mapped seed zones; n indicates the number of populations represented in each terminal node. Lower part of the figure shows spatial projections of seed zones for each subspecies group across the Intermountain Region of western North America. Left map shows zones for the branch containing ssp. *brevifolius* and ssp. *californicus* (abbreviated BC); right map, ssp. *elymoides* and ssp. *hordeoides* (abbreviated EH). Mapped area is defined by EPA Level III Ecoregions (Omernik and Griffith 2014; Figure 1), omitting areas beyond 1 standard deviation of the sampled climate range.

When subspecies identity was included as a predictor variable in regression tree models, it was the first variable identified for partitioning and accounted for 21% of the variance (Figure 7). Populations belonging to *E. elymoides ssp. brevifolius* and ssp. *californicus*, plus three populations not identified to subspecies, came together in one side of the primary partition, labeled ‘BC’, opposite the group ‘EH’ comprised of ssp. *elymoides*, ssp. *hordeoides*, and seven other unidentified populations (Figure 7). Subsequent partitions were determined by climate variables, especially variables measuring temperature or degree‐days (Figure 7), and accounted for an additional 27% of model variance, leaving 52% error variance. The BC group was subdivided into 4 secondary groups, one of which was further partitioned into two tertiary groups, leading to 5 terminal nodes (Figure 7). The EH group, with 3 secondary groups and 5 terminal nodes, had greater population representation but lower variance than the BC group (Figure 7).

Separate zone maps were developed for the BC and EH groups by projecting their respective secondary and tertiary subdivisions onto the mapping region (Figure 7). These maps showed that zonation patterns based on trait‐climate associations were similar in some respects for both groups of subspecies, even though zone divisions and boundaries were not identical (Figure 7). Both groups exhibited zones that were aligned with both elevation gradients and the east–west gradient of seasonal temperature difference, although the east–west division was more distinct for the BC group and elevational zonation was more finely subdivided for the EH group (Figure 7).

## 4 Discussion

### 4.1 Trait Variation and Its Climatic Associations

In this study, we detected significant trait variation among *Elymus elymoides* populations that was associated with climatic variation at population source locations, consistent with local adaptation expected in this region (Baughman et al. 2019). Plants from cooler and wetter climates generally grew larger and had later flowering phenology than plants from warmer and drier climates. Similar associations between these traits and temperature and aridity gradients, as well as seasonal temperature difference, have been found in most other Intermountain Region grass species that have been studied, including *Achnatherum hymenoides* (Roem. & Schult.) Barkworth (Johnson et al. 2012), *Pseudoroegneria spicata* (Pursh) Á. Löve (St. Clair et al. 2013), *Poa secunda* J. Presl (Johnson et al. 2015), *Leymus cinereus* (Scribn. & Merr.) Á. Löve (Johnson and Vance‐Borland 2016), and *Achnatherum thurberianum* (Piper) Barkworth (Johnson et al. 2017), and are likely evidence of similar responses to common selection gradients. This finding also agrees with studies of *E. elymoides* by Clary (1975), who tested several size traits and initiation of anthesis against source climate, and by Blumenthal et al. (2021), who tested juvenile biomass against source climate. Larger plants with later flowering phenologies also originated from milder, less continental climates with a combination of lower seasonal temperature difference and long growing seasons, found primarily on the western side of our study region. These climates were not sampled by Clary (1975), but this association with milder climates was also found in Blumenthal et al. (2021). We found that narrow leaf widths were related to aridity, which was also noted by Clary (1979) and is similar to some patterns found by Blumenthal et al. (2021).

In some cases, trait differences were related to subspecies identity. Differences between ssp. *elymoides* and *brevifolius* in size and phenology were similar to those found in Jones et al. (2003), with ssp. *brevifolius* having higher biomass and later heading date than ssp. *elymoides*. While leaf traits were associated with aridity gradients generally, differences in these traits between subspecies were not pronounced in our study. This contrasts with Jones et al. (2003), who found that ssp. *elymoides* had narrower and smaller leaves than ssp. *brevifolius*, and that ssp. *elymoides* tends to be found in warmer and drier climates than ssp. *brevifolius*. Parsons, Jones, Larson, et al. (2011); Parsons, Jones, and Monaco (2011) found a general pattern of lower biomass, earlier phenology, and narrower leaves with higher aridity for ssp. *brevifolius*, similar to our findings, indicating that significant trait‐climate associations exist within subspecies. When we assessed subspecies identity in a regression tree model, it was the most important partition, with two groups (BC: ssp. *brevifolius* + ssp. *californicus* and EH: ssp. *elymoides* + ssp. *hordeoides*). This indicates that the subspecies within each group have either experienced similar selective regimes and possible trait convergence, or that they share a lineage that has evolved distinct characteristics. Genetic analysis supports the monophyletic nature of *E. elymoides ssp. elymoides* with regard to other subspecies (Larson et al. 2003; Parsons, Jones, Larson, et al. 2011), while ssp. *brevifolius* has been shown to be paraphyletic with ssp. *californicus* (Parsons, Jones, Larson, et al. 2011). No genetic information is available for ssp. *hordeoides* in the Intermountain Region (Parsons, Jones, Larson, et al. 2011). Together, this suggests that within‐group similarities are likely to be reflective of shared genetic and demographic histories rather than convergent evolution among less‐related types.

### 4.2 Plasticity in Trait Expression Across Gardens

While there were strong signals of trait associations with climate at population source locations, the common garden growth environment also had a strong effect on trait expression in *E. elymoides*, explaining more trait variation than source location. Our findings broadly agree with the range of trait variation found across other published studies on *E. elymoides* , which were generally conducted in different locations and/or years (Clary 1975, 1979; Jones et al. 2003; Parsons, Jones, Larson, et al. 2011; Parsons, Jones, and Monaco 2011; Blumenthal et al. 2021), though none of these studies specifically measured or analyzed the effects of growth environment on trait expression. Variation in population‐level trait expression across gardens also differed by source location. Taken together, this indicates that the *E. elymoides* phenotypes demonstrate significant plasticity in environmental response, with the degree of plasticity partly explained by climate at source locations. These levels of trait plasticity are comparable with those found in the relatively plastic *P. secunda* (Espeland et al. 2018), and greater than some other Intermountain Region grasses (e.g., St. Clair et al. 2013).

Because trait plasticity can be adaptive in variable environments, restoration success might depend on selecting sources with the type of plasticity best suited to specific restoration site environments. For example, Espeland et al. (2018) found that *P. secunda* populations from more extreme climates demonstrated plasticity in some traits (phenology, panicle number, and biomass) while populations from milder climates demonstrated plasticity in other traits (leaf size, panicle length, plant habit, and survival), with populations from extreme climates demonstrating more trait plasticity overall. While our data show that *E. elymoides* populations from milder climates were generally more plastic, we also found differential patterns in trait plasticity based on source climate. Generally, populations from milder climates (wetter with lower seasonal temperature difference) demonstrated the most trait plasticity, particularly for morphological characteristics, and populations from the most extreme climates (warmer and drier) demonstrated the least, with one exception, maturation date, which showed the opposite pattern. These patterns suggest that different climatic conditions may have selected for or against plasticity in different traits. Hypothetically, plasticity in growth rates and other morphological traits might be advantageous in milder climates in years when growing conditions are particularly favorable, but might be detrimental in more extreme climates where inaccurate response to early growth cues may lead to exposure to late season droughts. Whereas, plasticity in seed maturation might allow for a response to cues related to the timing of late season drought, and thus escape from potential late‐season losses to seed production in climates prone to such conditions.

### 4.3 Fixed‐Boundary Seed Zones

The geographic coverage of the *E. elymoides* seed zones follows the patterns described above for trait‐climate associations. In particular, A zones encompass milder climates in the western part of the range; B zones encompass cooler and wetter parts of the continental interior, which are generally at higher elevations; and C zones encompass warmer and drier areas of the continental interior, which are generally at lower elevations. These patterns are broadly similar to the other Intermountain Region grasses for which empirical seed zones have been constructed from genecological provenance studies using standard modeling procedures. While none of the Intermountain Region grass seed zones perfectly overlap, consistent patterns are present. For example, the five grasses ( *A. thurberianum* , *E. elymoides* , *L. cinereus* , *P. secunda* , *P. spicata* ) with seed zones in the Snake River Plain ecoregion all have distinct zones between the upper and lower Snake River Plain (see St. Clair et al. 2013; Johnson et al. 2015, 2017; Johnson and Vance‐Borland 2016).

Differences in trait‐climate associations between subspecies groups suggest that taxonomic distinctions could be important to seed transfer decisions for *E. elymoides* . While some species have genetic lineages that are geographically distinct and can be mapped easily onto species‐level seed zones (e.g., Massatti and Winkler 2022), *E. elymoides* subspecies groups have overlapping ranges, which prevents straightforward mapping (Massatti et al. 2025). Separate seed transfer models corresponding to each subspecies group, which we have provided, would be needed to take subspecies distinctions into account. However, there are some additional factors related to *E. elymoides* that could affect the usefulness of separate models. Most importantly, it is difficult for most seed collectors to distinguish *E. elymoides* subspecies in the field, and accurate identifications often require significant botanical expertise. Additionally, in contrast to other systems such as *L. cinereus* (Johnson and Vance‐Borland 2016), which demonstrates widespread ploidy variation, risk to the genetic integrity of remnant populations from outbreeding with seeded populations is low, due to a lack of regional ploidy variation and a high self‐pollination rate (Barkworth et al. 1983). Therefore, it is reasonable for restoration practitioners to use seed zones developed for the entire species, particularly for guiding initial wildland collections, or in cases when wildland seed is being used directly in restoration. That said, projects with longer time horizons, such as seed increase and germplasm development and release approaches, which are common in the Intermountain Region (Jones 2019; Baughman et al. 2022), might benefit from guidance based on seed transfer models at the subspecies level.

Trait plasticity is an important consideration in seed transfer guidance and is of growing interest to restoration practitioners (Vitt et al. 2022; Leites and Garzόn 2023). However, trait plasticity is difficult to incorporate directly into seed transfer models because the precision of population‐level plasticity estimates depends on the number of environments that are tested, with ten or more test environments often required to approach the highest levels of precision (Wang et al. 2010). While we don't have enough data from our study for robust incorporation of trait plasticity into our seed transfer models, and therefore it is not incorporated, the information that we present here can still be useful in seed‐sourcing decisions for *E. elymoides* . A couple of points stand out: first, the generally high levels of trait plasticity demonstrated in *E. elymoides* suggest that sources could be used across broader seed zones, defined at higher levels of the regression tree hierarchy, such as the A, B and C zones presented here; second, the association between trait plasticity and source climate follows the same climatic gradients that underpin our seed zones, reinforcing their potential efficacy.

### 4.4 Approaches for Modeling Seed Transfer

Our modeling approach partially followed previously developed methodology, in which composite trait axes obtained through dimensionality reduction techniques (e.g., PCA) serve as the response variables for transfer functions, which are then used to define seed zones. However, there are important differences between our approach to using PC trait axes in seed transfer model development and how they are used in more standard approaches, particularly in how fixed‐boundary seed zones are delineated. Standard approaches for developing fixed‐boundary seed zones select a set of PC axes with the highest explained variance, usually the top three, and often drop axes with less explanatory power, even if they are statistically significant. A segment partitioning method is then applied to each selected PC axis, such as equal intervals (e.g., St. Clair et al. 2013) or confidence intervals (e.g., Richardson and Chaney 2018). Each set of PC axis segments is then spatially projected separately, based on the specific trait‐climate associations for that axis, and then all projections are overlaid and intersected to form the final set of seed zones. Axis importance weighting comes into the procedure through generating a higher number of segments for PC axes with higher explanatory power. But this process is necessarily imprecise, because each PC axis is usually allowed a few discrete segments which can only roughly approximate the actual distribution of explanatory power across axes. In contrast, regression trees take advantage of the continuous scaling that is already present in PC axis values, which automatically weights each axis according to its importance relative to all other axes. Additionally, regression trees define trait‐climate associations based on information from all input PC axes at once, rather than separately for each axis, allowing climatic partitions to be ranked and projected based on their importance within the entire dataset. This allows for both greater precision and the ability to use all significant PC axes, maximizing information usage and reducing potential subjectivity in axis selection and segment partitioning; also making our method more replicable and potentially more automatable.

Because of trade‐offs between reducing the probability of maladaptation and the practical realities of seed zone implementation, questions regarding the appropriate number of seed zones can be complex. With standard approaches, subdividing or combining seed zones is not a straightforward process, usually requiring new decisions on PC trait axis partitioning and subsequent reprojection, and relevant details are often not provided in published studies (Johnson and Vance‐Borland 2016). An advantage of regression tree modeling is that it provides an explicit hierarchical structure that relates directly to seed zone geographies. Therefore, with the information provided here, any practitioner could move up and down the pre‐constructed hierarchy to subdivide or combine seed zones in a way that still considers climatic adaptation. For example, we have noted that information regarding trait plasticity could be used to justify using fewer and broader seed zones in *E. elymoides* .

Transfer functions are useful in predictive modeling of population trait values across varying geographies and climates. The standard procedure for developing seed transfer functions is to use multiple linear and polynomial regression, whereas we use random forests here. Linear and polynomial parameters can be interpreted in a straightforward manner when relationships are genuinely linear or polynomial (Kilkenny 2015; Richardson and Chaney 2018; St. Clair et al. 2022). However, linear and polynomial parameters can oversimplify trait‐climate associations in cases where these relationships are complex. In contrast, random forest modeling is sensitive to complex trait‐climate associations, such as abrupt thresholds or non‐continuous distributions, demonstrated here by several *E. elymoides* trait‐climate associations, and can generate better representations of actual trait‐climate associations even when the potential for model over‐fitting is considered (e.g., Grömping 2009). Because it is possible that real‐world trait‐climate associations are more complex than would be predicted by standard approaches, the use of random forest models could help increase basic understanding of biological adaptations to climate (Fitzpatrick and Hargrove 2009; Chmura et al. 2019), and may support seed transfer models that are more effective at predicting how plants will respond to ongoing climate change (e.g., Anderson and Wadgymar 2020).

Transfer functions are also ideal for developing focal‐point seed transfer models, which do away with zone delineation and dynamically generate suitability estimations for potential source‐site matches based on user‐specified locations (source and/or site) (Shryock et al. 2018; St. Clair et al. 2022). Recently, there has been movement to increase the use of focal‐point seed transfer models based on their greater precision, flexibility, and capacity to integrate future climate projections into seed‐sourcing decisions (Kilkenny 2015; Richardson and Chaney 2018). However, focal‐point seed transfer models can be more difficult to implement in practice than fixed‐boundary seed zones. Due to practical constraints of seed collection and production, focal‐point seed transfer models are generally only implemented in cases when restoration locations are already known based on planned disturbances, such as mine reclamation or post‐logging rehabilitation (Ying and Yanchuk 2006), or in cases where wildland seed collections are of sufficient quality, quantity, and availability to support restoration of areas affected by unplanned disturbances (e.g., Richardson and Chaney 2018). Fixed‐boundary seed zones tend to be easier to implement in practice, though less precise (Rehfeldt 1986), and are likely to be necessary when sufficient seed quantities must be produced through agronomic methods—a process that requires significant effort and long ramp‐up times, favoring seed that can be used across large areas (Erickson and Halford 2020)—and in cases where restoration is reactive and has short implementation windows requiring the use of immediately available seed, both of which are the case for most post‐wildfire restoration in the Intermountain Region (Barga et al. 2023; but see Richardson and Chaney 2018 for an exception). Ultimately, this argues for the continued use of both fixed‐boundary and focal‐point seed transfer models, and therefore the need for modeling approaches that can produce both output types.

### 4.5 Seed Transfer Modeling in Changing Climates

Given the rapid rate of climate change, a question arises with important consequences for how seed transfer guidelines are developed and used: should seed sources be matched to restoration sites based on current or future climates? This is a heated topic with many strident viewpoints (e.g., McKone and Hernández 2021; Beck et al. 2025), even though current evidence is likely insufficient to support universal adoption of any proposed strategy (Breed et al. 2018; Vitt et al. 2022). Proponents of using seed sources adapted to future climates argue that many plant populations lack the capacity to migrate or evolve at the current rate of climate change (Aitken et al. 2008; Breed et al. 2013; Anderson and Wadgymar 2020), let alone potential future rates, and that seed transfer based on future climates would introduce additional genetic variation and climate‐adapted alleles that could support rapid adaptation and delay population extirpation (Broadhurst et al. 2008; Aitken and Whitlock 2013; Prober et al. 2015; Havens et al. 2015; Hoffmann et al. 2021). In contrast, proponents of using seed sources adapted to current conditions point out that plant populations often evolve more rapidly than is generally believed and may be able to track changing climates (Kulbaba et al. 2019; Torres‐Martínez et al. 2019); that significant uncertainty surrounds future climate projections, including the likelihood that some climates will have no contemporary analogs (Williams and Jackson 2007; Soley‐Guardia et al. 2024); and that long distance seed transfers could lead to genetic and demographic disruptions to local populations and interference with other ecological processes and relationships, such as pollinator networks (Bucharova et al. 2021; Hulting et al. 2024).

We acknowledge that these are complex and critical issues that require significant thought when making seed‐sourcing decisions, including the recognition that all seed transfer actions carry risks (Jordan et al. 2024) and that nuanced approaches are possible (Bucharova et al. 2019; Török et al. 2024). While a specific stance in this debate is beyond the scope of the current study—the choice to focus on current climates is based on stakeholder needs and policies, rather than larger philosophical considerations—the seed transfer modeling approaches presented here can be used with any current or future climate scenarios, supporting evidence‐based decision frameworks and allowing for practitioner choice. Focal‐point seed transfer models, regardless of the method used to produce them, inherently lend themselves to dynamic reprojection into any chosen climate scenario, and this capability has been incorporated in publicly available tools (Shryock et al. 2018; St. Clair et al. 2022; Silva et al. 2025). Fixed‐boundary seed zones can also incorporate future climate scenarios, but must be reprojected and made available by their developers when using standard modeling procedures (e.g., Thomson et al. 2010; Richardson and Chaney 2018). Because zone partitioning processes can be automated, regression tree modeling has the potential to support dynamic reprojections of fixed‐boundary seed zones based on user‐selected climate scenarios, but to our knowledge, has not been implemented in this way in publicly available tools, suggesting a path for future work.

## Funding

This work was supported by Great Basin Native Plant Project, USDI Bureau of Land Management, USDA Forest Service Pacific Northwest Research Station, Rocky Mountain Research Station, Deschutes National Forest.

## Conflicts of Interest

The authors declare no conflicts of interest.

## Supporting information


**Figure S1:** Results of variance components analysis for traits of 98 *Elymus elymoides* populations.

**Figure S2:** Trait plasticity by zone for 98 populations of *Elymus elymoides* grown at three common gardens.

**Figure S3:** Trait values by zone for 98 populations of *Elymus elymoides* grown at three common gardens.

**Figure S4:** Scaled trait values for each partition of a regression tree model built from principal component axes of trait values of *Elymus elmoides*.

**Table S1:** Climate variables (30‐year normal, 1981–2010) used for modeling and mapping.
**Table S2:** Effects of collection year and seedling greenhouse time on subsequent trait expression in *E. elymoides*.
**Table S3:** Annual precipitation and temperature values at common gardens during the three study years.
**Table S4:** Loadings of *Elymus elmoides* trait ×garden variables (rows) on 6 significant principal component axes.
**Table S5:** Variable importance (average decrease in residual sum of squares) for random forest models.

## Data Availability

The data that supports the findings of this study are available at the USDA Forest Service Research Data Archive: https://www.fs.usda.gov/rds/archive/.
