# Supplementary material for: Modeling Phenotypic Trait Variation and Plasticity in Elymus elymoides to Guide Climate‐Informed Seed Transfer
Source: Evol Appl. 2026 Mar 6;19(3):e70211. doi: 10.1111/eva.70211 (PMC12965906; doi:10.1111/eva.70211)
Supplement: Supplementary file 1 — Figure S1: Results of variance components analysis for traits of 98 Elymus elymoides populations. [file EVA-19-e70211-s004.pdf]

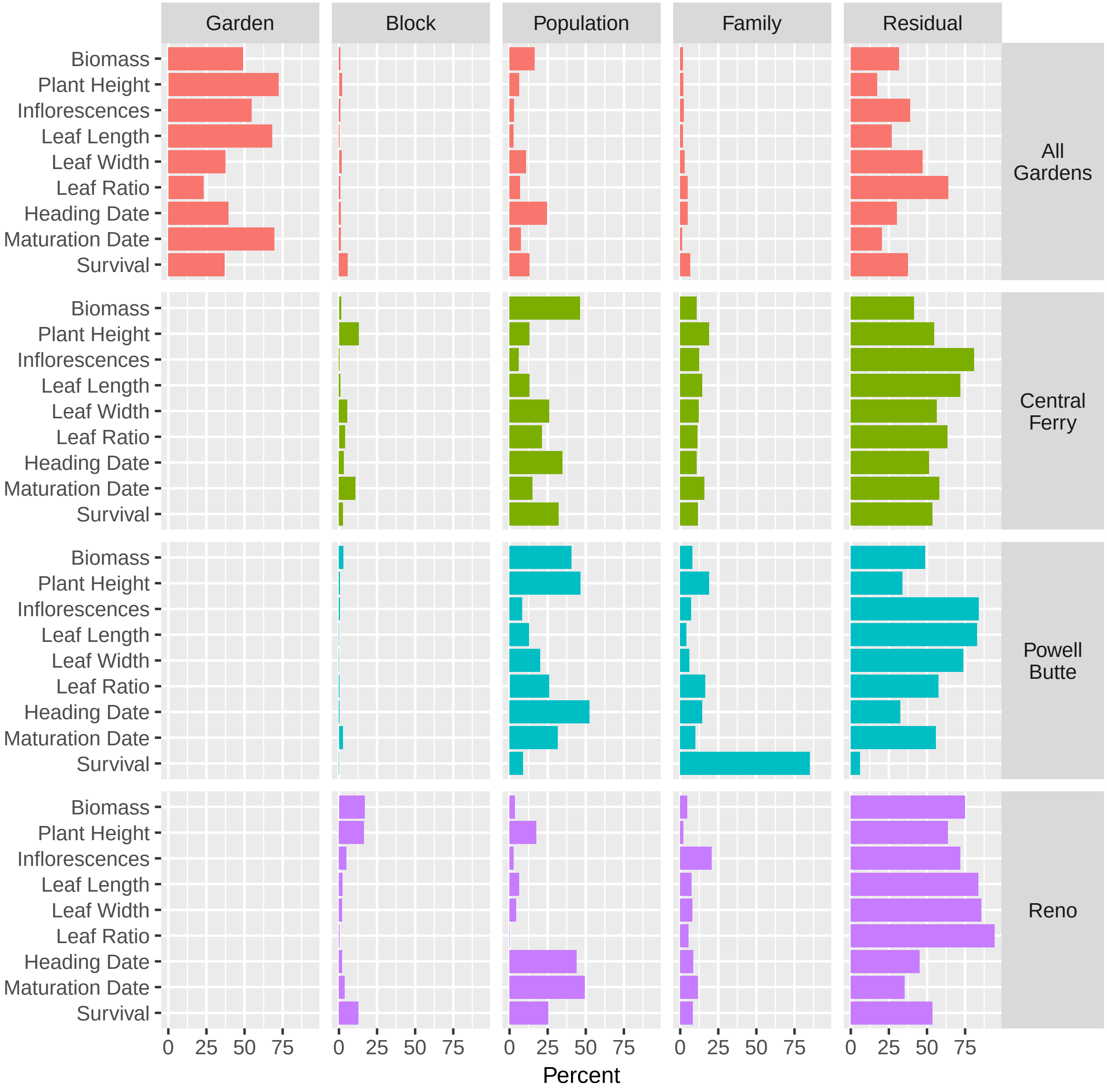

Figure S1. Results of variance components analysis for traits of 98 *Elymus elymoides* populations measured at three common gardens. Values shown are percent variance attributed to garden, block within garden, population, family within population, and residual error. Analyses carried out for all gardens (top panel; see also Table 3) were repeated for each individual garden (lower three panels).
