# Supplementary material for: Modeling Phenotypic Trait Variation and Plasticity in Elymus elymoides to Guide Climate‐Informed Seed Transfer
Source: Evol Appl. 2026 Mar 6;19(3):e70211. doi: 10.1111/eva.70211 (PMC12965906; doi:10.1111/eva.70211)
Supplement: Supplementary file 2 — Figure S2: Trait plasticity by zone for 98 populations of Elymus elymoides grown at three common gardens. [file EVA-19-e70211-s003.pdf]

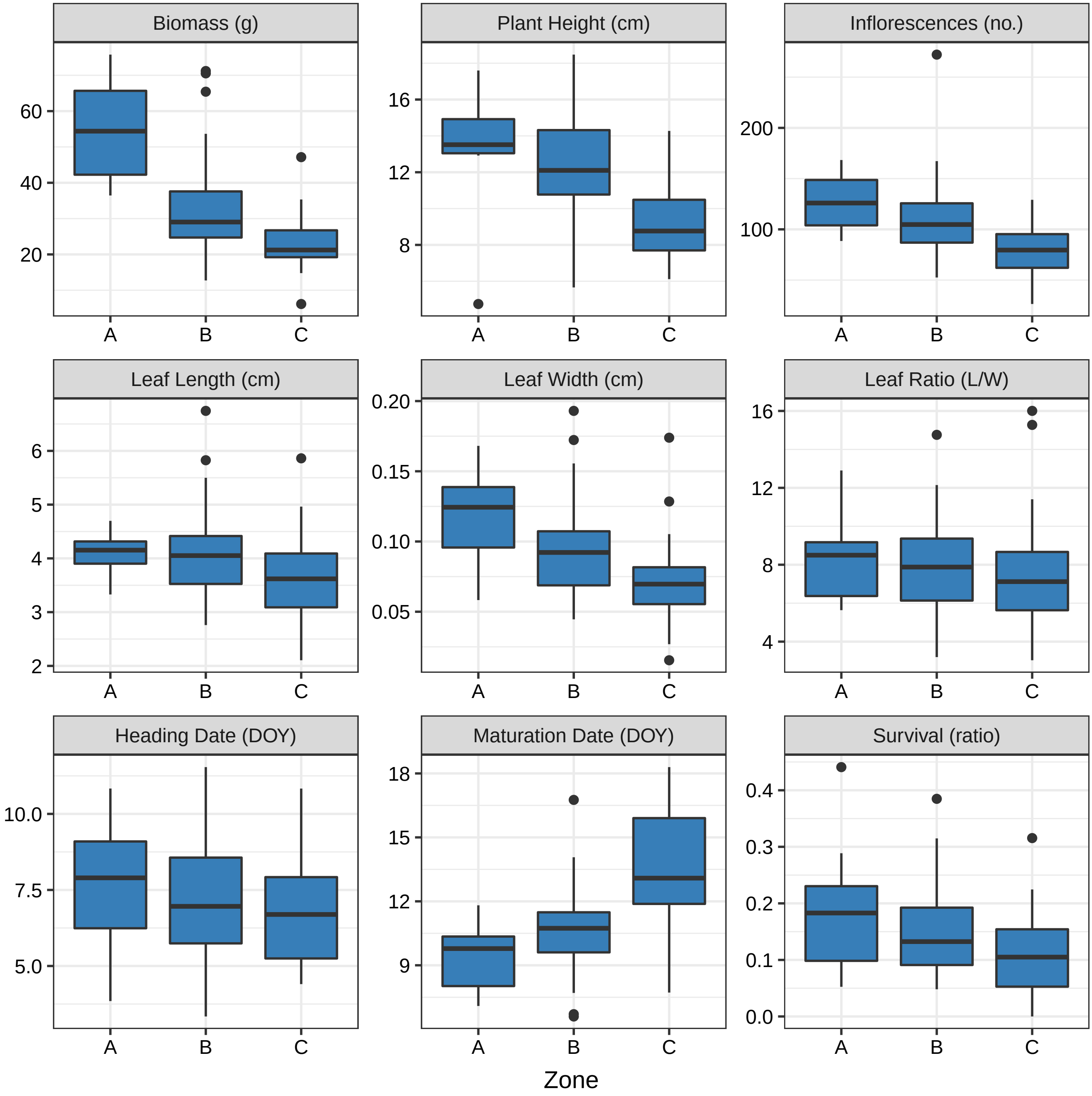

Figure S2. Trait plasticity by zone for 98 populations of *Elymus elymoides* grown at three common gardens. Plasticity was measured as the standard deviation of mean measured trait values for a given population across gardens. Boxplots show the distribution of standard deviations for populations in the three primary seed transfer zones shown in Figure 3. The climate of Zone A is marked by lower seasonal temperature difference than Zones B and C, and Zone B is cooler and generally wetter than Zone C.
