# Supplementary material for: Modeling Phenotypic Trait Variation and Plasticity in Elymus elymoides to Guide Climate‐Informed Seed Transfer
Source: Evol Appl. 2026 Mar 6;19(3):e70211. doi: 10.1111/eva.70211 (PMC12965906; doi:10.1111/eva.70211)
Supplement: Supplementary file 3 — Figure S3: Trait values by zone for 98 populations of Elymus elymoides grown at three common gardens. [file EVA-19-e70211-s005.pdf]

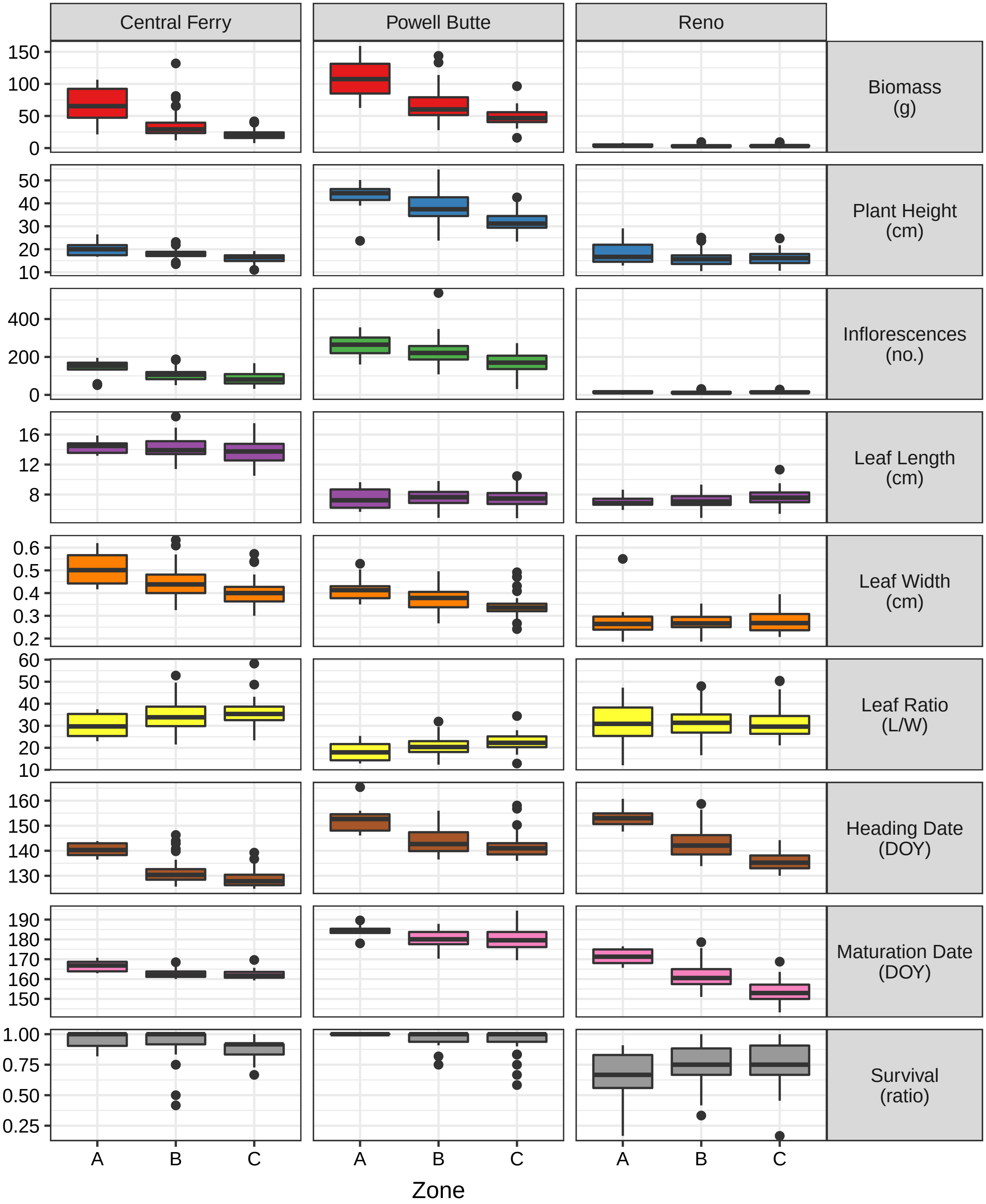

Figure S3. Trait values by zone for 98 populations of *Elymus elymoides* grown at three common gardens. Boxplots show the distribution of trait values for populations in the three primary seed transfer zones shown in Figure 3. The climate of Zone A is marked by lower seasonal temperature difference than Zones B and C, and Zone B is cooler and generally wetter than Zone C.
