# Supplementary material for: Modeling Phenotypic Trait Variation and Plasticity in Elymus elymoides to Guide Climate‐Informed Seed Transfer
Source: Evol Appl. 2026 Mar 6;19(3):e70211. doi: 10.1111/eva.70211 (PMC12965906; doi:10.1111/eva.70211)
Supplement: Supplementary file 4 — Figure S4: Scaled trait values for each partition of a regression tree model built from principal component axes of trait values of Elymus elmoides. [file EVA-19-e70211-s002.pdf]

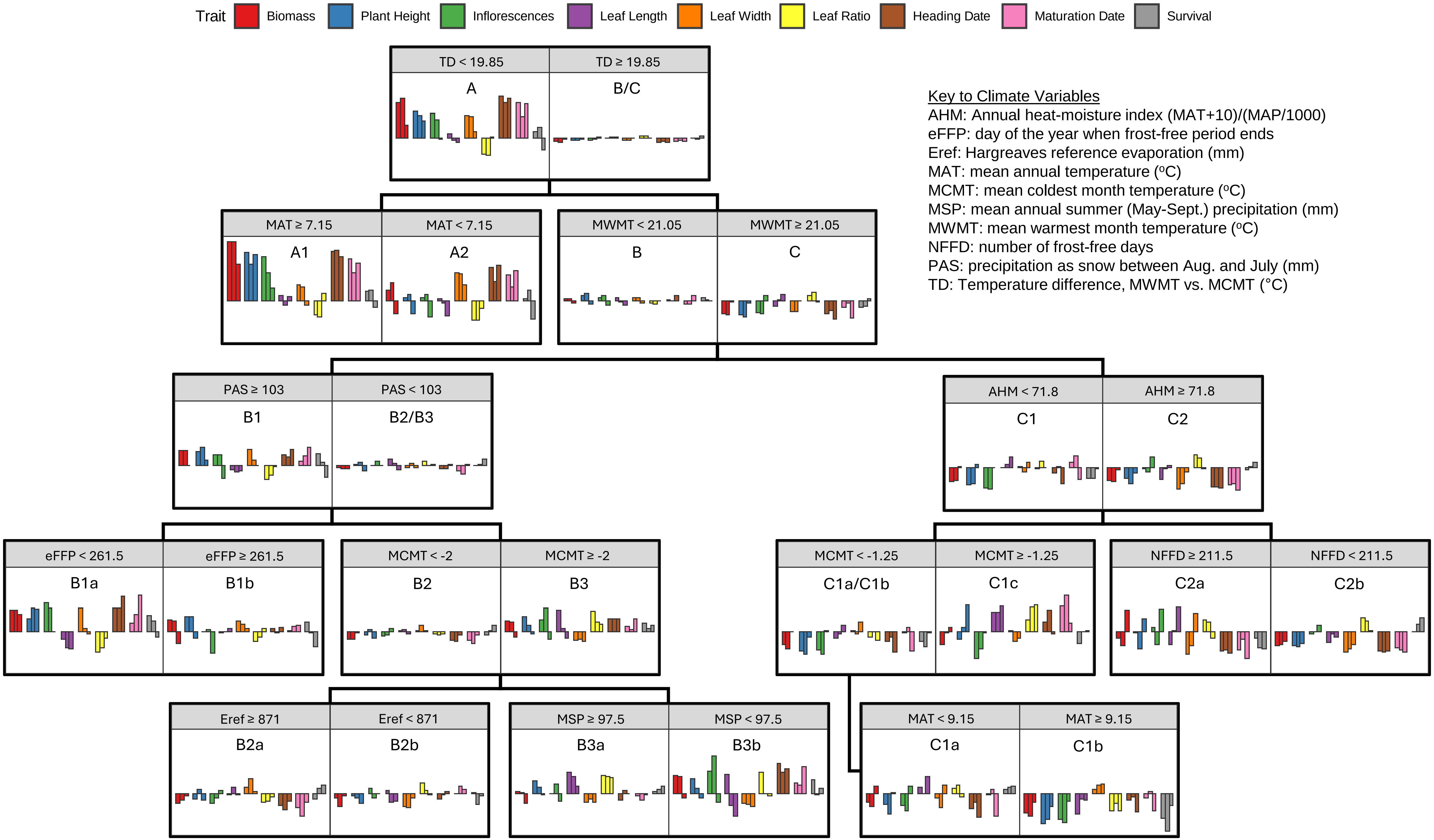

Figure S4. Scaled trait values for each partition of a regression tree model built from principal component axes of trait values of *Elymus elmoides* plants grown in three common gardens. Size and direction of bars denote relative values, centered and scaled independently for each trait and garden and averaged at each level and partition of the tree hierarchy. Letter/number codes in cells correspond to seed transfer zones; see Figure 3.
