# Supplementary material for: Modeling Phenotypic Trait Variation and Plasticity in Elymus elymoides to Guide Climate‐Informed Seed Transfer
Source: Evol Appl. 2026 Mar 6;19(3):e70211. doi: 10.1111/eva.70211 (PMC12965906; doi:10.1111/eva.70211)
Supplement: Supplementary file 5 — Table S1: Climate variables (30‐year normal, 1981–2010) used for modeling and mapping. Table S2: Effects of collection year and seedling greenhouse time on subsequent trait expression in E. elymoides. Table S3: Annual precipitation and temperature values at common gardens during the three study years. Table S4: Loadings of Elymus elmoides trait ×garden variables (rows) on 6 significant principal component axes. Table S5: Variable importance (average decrease in residual sum of squares) for random forest models. [file EVA-19-e70211-s001.pdf]

Table S1. Climate variables (30-year normals, 1981-2010) used for modeling and mapping genetic trait variation of *Elymus elymoides* in relation to climate in the Intermountain Region. Source: ClimateWNA (Wang et al. 2016).

**Precipitation**	
MAP	Mean annual precipitation (mm)
MSP	Mean annual summer (May to Sept.) precipitation (mm)
PAS	Precipitation as snow between Aug. and July (mm)
RH	Mean annual relative humidity (%)
**Temperature**	
EMT	Extreme minimum temperature over 30 years (°C)
EXT	Extreme maximum temperature over 30 years (°C)
MAT	Mean annual temperature (°C)
MCMT	Mean coldest month temperature (°C)
MWMT	Mean warmest month temperature (°C)
TD	Temperature difference between MWMT and MCMT (°C)
**Degree-days**	
DD_0	Degree-days below 0°C, chilling degree-days
DD_18	Degree-days below 18°C, heating degree-days
DD5	Degree-days above 5°C, growing degree-days
DD18	Degree-days above 18°C, cooling degree-days
**Frost**	
NFFD	Number of frost-free days
FFP	Frost-free period (consecutive days)
bFFP	Day of the year on which FFP begins
eFFP	Day of the year on which FFP ends
**Aridity**	
AHM	Annual heat-moisture index (MAT+10)/(MAP/1000))
SHM	Summer heat-moisture index ((MWMT)/(MSP/1000))
CMD	Hargreaves climatic moisture deficit (mm)
Eref	Hargreaves reference evaporation (mm)
**Solar Radiation**	
MAR	Mean annual solar radiation (MJ m^{-2} d^{-1})

Table S2. Effects of collection year and seedling greenhouse time on subsequent trait expression of *Elymus elymoides* plants at three common gardens. Mean trait values of populations measured at each garden served as data points for correlation analyses. Pearson correlation coefficients are shown if statistically significant (P < 0.05), with P values adjusted for multiple tests using the false discovery rate (Benjamini and Hochberg 1995).

Trait	Garden	Collection Year	Days in Greenhouse
Biomass	Central Ferry	-0.26	-0.24
Biomass	Powell Butte	-0.35	-0.24
Biomass	Reno	--	--
Plant Height	Central Ferry	--	--
Plant Height	Powell Butte	-0.24	--
Plant Height	Reno	--	--
Inflorescences	Central Ferry	--	--
Inflorescences	Powell Butte	--	--
Inflorescences	Reno	--	--
Leaf Length	Central Ferry	--	--
Leaf Length	Powell Butte	--	--
Leaf Length	Reno	--	--
Leaf Width	Central Ferry	-0.34	--
Leaf Width	Powell Butte	--	--
Leaf Width	Reno	--	--
Leaf Ratio	Central Ferry	--	--
Leaf Ratio	Powell Butte	--	--
Leaf Ratio	Reno	--	--
Heading Date	Central Ferry	-0.35	-0.26
Heading Date	Powell Butte	-0.38	--
Heading Date	Reno	-0.33	--
Maturation Date	Central Ferry	-0.31	--
Maturation Date	Powell Butte	--	--
Maturation Date	Reno	-0.34	--
Survival	Central Ferry	--	--
Survival	Powell Butte	--	--
Survival	Reno	--	0.32

Table S3. Annual precipitation and temperature values at common gardens during the three years from outplanting (2011) through trait measurement (2013) of *Elymus elymoides* populations. Variables are mean annual preciptation (MAP), mean summer precipitation (MSP), precipitation as snow (PAS), mean annual temperature (MAT), mean coldest month temperature (MCMT), mean warmest month temperature (MWMT), and temperature differential (TD), extracted from ClimateWNA (Wang et al. 2016).

	2011			2012			2013		
Variable	Central Ferry	Powell Butte	Reno	Central Ferry	Powell Butte	Reno	Central Ferry	Powell Butte	Reno
MAP (mm)	358	261	157	412	316	173	257	196	127
MSP (mm)	91	105	54	72	72	15	103	117	76
PAS (mm)	24	38	14	13	25	5	12	16	8
MAT (°C)	11.4	7.6	11.6	12.6	8.6	13.3	12.1	8.2	12.2
MCMT (°C)	1.9	-0.6	0.8	2.2	0.5	2.5	0.1	-2.3	-1.7
MWMT (°C)	23.4	17.6	24.2	24.7	18.3	25.7	25.7	19.9	26.8
TD (°C)	21.5	18.2	23.4	22.5	17.8	23.2	25.6	22.2	28.4

Table S4. Loadings of *Elymus elmoides* trait ×garden variables (rows) on 6 significant principal component axes (columns). Traits were measured at three common gardens. Color shading is on a scale from red (higher values) to blue (lower values).

Trait	Garden	Axis 1	Axis 2	Axis 3	Axis 4	Axis 5	Axis 6
Biomass	Central Ferry	-0.293	0.069	0.013	-0.132	0.073	-0.291
Biomass	Powell Butte	-0.312	0.120	0.045	-0.110	-0.053	-0.046
Biomass	Reno	-0.127	0.043	-0.440	-0.268	0.125	0.152
Plant height	Central Ferry	-0.253	0.179	-0.054	-0.111	0.018	-0.318
Plant height	Powell Butte	-0.239	0.066	-0.043	-0.125	-0.242	0.100
Plant height	Reno	-0.184	0.040	-0.404	-0.151	0.111	0.055
Inflorescences	Central Ferry	-0.220	0.115	0.121	-0.266	0.189	-0.321
Inflorescences	Powell Butte	-0.209	0.207	0.222	-0.218	-0.044	-0.011
Inflorescences	Reno	0.014	0.098	-0.305	-0.377	0.034	0.377
Leaf length	Central Ferry	-0.108	0.248	-0.106	0.317	-0.162	-0.140
Leaf length	Powell Butte	0.029	0.231	-0.196	0.199	-0.460	-0.086
Leaf length	Reno	0.044	0.157	-0.435	0.088	-0.156	-0.218
Leaf width	Central Ferry	-0.184	-0.323	-0.082	-0.013	-0.279	-0.027
Leaf width	Powell Butte	-0.094	-0.293	-0.079	-0.010	-0.477	-0.055
Leaf width	Reno	-0.002	-0.298	-0.231	-0.013	0.016	-0.431
Leaf ratio	Central Ferry	0.088	0.400	0.020	0.197	0.167	-0.086
Leaf ratio	Powell Butte	0.088	0.419	-0.093	0.183	0.019	-0.050
Heading date	Central Ferry	-0.308	-0.036	0.034	0.144	0.074	0.186
Heading date	Powell Butte	-0.278	-0.067	-0.051	0.276	0.105	0.109
Heading date	Reno	-0.305	-0.052	0.200	0.149	-0.006	0.020
Maturation date	Central Ferry	-0.238	-0.008	-0.068	0.220	-0.007	0.346
Maturation date	Powell Butte	-0.205	-0.022	-0.189	0.292	0.237	0.119
Maturation date	Reno	-0.310	-0.013	0.132	0.165	-0.028	0.063
Survival	Central Ferry	-0.086	0.181	0.127	-0.218	-0.039	0.102
Survival	Powell Butte	-0.074	0.141	0.204	-0.187	-0.324	0.023
Survival	Reno	0.093	0.242	0.072	-0.068	-0.303	0.246

Table S5. Variable importance (average decrease in residual sum of squares) for random forest models predicting trait variation of *Elymus elymoides* populations. Rows are climate predictor variables (see Table S1) and columns are significant principal component axes of traits measured at three common gardens. Color shading from red (higher values) to blue (lower values) is scaled individually for each column.

	Axis 1	Axis 2	Axis 3	Axis 4	Axis 5	Axis 6
MAP	39.31	28.30	13.99	12.26	7.87	7.44
MSP	12.35	15.41	11.08	8.27	7.54	2.72
PAS	36.60	21.98	9.79	9.42	4.93	4.91
RH	7.89	9.15	4.49	6.36	7.78	9.38
EMT	28.12	12.16	7.87	11.45	10.63	3.41
EXT	15.93	11.08	13.74	9.84	5.92	6.62
MAT	14.46	7.94	8.10	6.77	5.14	2.08
MCMT	56.29	15.64	12.55	10.46	11.51	4.39
MWMT	73.25	10.44	8.85	7.82	6.11	5.39
TD	123.94	10.78	9.91	8.06	7.65	5.03
DD_0	37.00	32.72	18.13	10.51	9.43	4.58
DD_18	23.60	12.51	22.77	11.36	7.58	4.12
DD5	35.37	9.56	13.98	11.84	4.76	5.37
DD18	61.01	9.94	9.01	9.06	5.23	5.72
NFFD	13.77	15.96	8.06	8.05	6.53	2.54
FFP	16.19	15.04	11.11	9.33	5.67	4.37
bFFP	18.18	10.71	14.31	10.56	5.01	5.68
eFFP	15.99	28.86	5.42	6.61	4.31	4.15
AHM	41.47	26.31	16.13	13.13	7.77	5.88
SHM	20.38	17.72	12.54	8.58	8.19	3.19
CMD	13.82	9.72	6.30	9.44	5.27	4.00
Eref	14.54	11.83	5.82	9.36	7.88	4.37
MAR	15.71	24.23	6.44	8.96	5.80	8.52
